# Efficient energy transfer mitigates parasitic light absorption in molecular charge-extraction layers for perovskite solar cells

**DOI:** 10.1038/s41467-020-19268-w

**Published:** 2020-11-02

**Authors:** Hannah J. Eggimann, Jay B. Patel, Michael B. Johnston, Laura M. Herz

**Affiliations:** Department of Physics, University of Oxford, Clarendon Laboratory, Parks Road, Oxford, OX1 3PU UK

**Keywords:** Devices for energy harvesting, Solar cells, Solar cells

## Abstract

Organic semiconductors are commonly used as charge-extraction layers in metal-halide perovskite solar cells. However, parasitic light absorption in the sun-facing front molecular layer, through which sun light must propagate before reaching the perovskite layer, may lower the power conversion efficiency of such devices. Here, we show that such losses may be eliminated through efficient excitation energy transfer from a photoexcited polymer layer to the underlying perovskite. Experimentally observed energy transfer between a range of different polymer films and a methylammonium lead iodide perovskite layer was used as basis for modelling the efficacy of the mechanism as a function of layer thickness, photoluminescence quantum efficiency and absorption coefficient of the organic polymer film. Our findings reveal that efficient energy transfer can be achieved for thin (≤10 nm) organic charge-extraction layers exhibiting high photoluminescence quantum efficiency. We further explore how the morphology of such thin polymer layers may be affected by interface formation with the perovskite.

## Abstract (web summary)

The performance of perovskite solar cells can be limited by light absorption loss in organic charge extraction layers, through which sun light must propagate before reaching the perovskite. Here, the authors demonstrate that efficient energy transfer to the perovskite layer from a thin organic layer is able to eliminate this parasitic loss.

## Introduction

Metal-halide perovskites (MHPs) offer great potential as active layers in solar cells owing to favourable optoelectronic properties, such as band-gap tunability^1,2^, high charge-carrier mobilities^3^, low exciton-binding energies^4^ and high defect tolerance^5^. Immense progress towards commercialisation has been made with lab-scale perovskite solar cells presently reaching efficiencies of over 25%^6^. One important challenge in this area is the optimisation of the charge-transport layers (CTLs)^7–9^. CTLs have become an essential component of perovskite device architectures because the facile migration of mobile ions within the MHP prevents reliable doping, meaning that the conventional p–n-junction approach may prove too difficult to accomplish for MHP layers^10,11^. Current state-of-the-art devices use organic semiconductors as CTLs that offer many benefits such as easy tailorability, low-cost and large-scale fabrication^9,12,13^ and surface passivation for significantly improved charge extraction at the electrodes^7,10^. A variety of different organic semiconductors, including *π*-conjugated polymers, are employed, particularly in multijunction MHP devices. Importantly, the currently common general architecture of these solar cells means that a CTL is usually the first semiconductor in the device to interact with light, because organic molecular CTLs are often deposited directly onto transparent electrodes on sun-facing substrates, followed by the light-harvesting perovskite layer^14–18^. One issue that has been highlighted in this context is parasitic absorption of light in the CTL, which can reduce device performance^19–23^ by lowering the amount of light that reaches the MHP to generate charge-separated electrons and holes, which is graphically depicted in Supplementary Fig. 1.

Such parasitic absorption of light by a sun-facing CTL is clearly detrimental, and may affect both front CTLs in single-junction devices, and those embedded amidst more complex tandem architectures. However, efforts to reduce such effects have to date mostly focused on simply choosing CTLs with a low absorption coefficient in the visible region of the spectrum. This approach severely restricts the choice of semiconductors available, which is undesirable, given that CTLs also need to comply with a large number of other requirements, including sufficiently high charge-carrier mobilities, and correct energetic band alignment with the conduction or the valence band of the active MHP layer. Interestingly, an additional material space may be opened if parasitic absorption by the organic semiconductor can be counteracted by subsequent energy transfer (ET) to the MHP. As part of this mechanism, light first creates a bound electron–hole pair (exciton) in the molecular CTL, whose oscillating electronic dipole moment then couples with the electronic transitions available in the MHP. Radiationless transfer may then occur from the CTL to the MHP, converting an exciton in the CTL into an electron–hole pair in the MHP, which is extracted as photocurrent through the respective CTLs. Such dipole–dipole interactions between an energy donor and an acceptor can be described within the theory of resonant ET, which was originally developed by Theodor Förster for ET within a molecular solution (3-dimensional space) with uniform donor and acceptor concentrations^24–26^. Such Förster-resonant ET operates over distances of up to 10–20 nm, comparable to typical CTL thicknesses, whereas the alternative mechanism of charge transfer is effective over much shorter distances only, and will therefore not be included in our discussions here^27^. It is also worth noting that in contrast to charge-transfer mechanisms, Förster-resonant ET occurs through space and is therefore independent of the quality of the interfaces encountered, e.g., at the CTL-perovskite heterojunction. Förster-resonant ET has become a widely used theory for measuring nanoscale distances, and has been extended to a wide range of applications and material geometries^28–34^. For the design of molecular CTLs in MHP solar cells, an understanding of the ET between the photoexcited organic semiconductor and the MHP layer is clearly of great importance; however, an in-depth investigation is still outstanding.

An additional consideration for the design of CTLs is how their molecular morphology is affected by a CTL:MHP interface. Effective functioning of CTLs relies on excellent charge-carrier transport through to the metal electrodes. In conjugated polymers, charge-carrier transport occurs either between neighbouring polymer chains with overlapping *π* orbitals (interchain) or along the polymer backbone (intrachain)^35^. Both transport pathways are sensitive to the arrangement of the polymer chains, which differs between polymers in solution and in thin films^36^, and is strongly influenced by film morphology^37–41^. In addition, chain morphology affects electronic energy levels, which may complicate their alignment with other conducting layers in the device^42^. Proper understanding of polymer structures within the transport layer, and in particular, at interfaces to other layers such as MHP films, is therefore critical for the development of efficiently working devices.

In this study, we establish the conditions required for efficient ET to occur between a thin, light-facing organic conjugated polymer layer and an underlying MHP film. To this end, we deposited thin films of four conjugated polymers commonly used in optoelectronic devices onto methylammonium lead iodide (MAPbI_3_) layers that had been thermally evaporated onto quartz substrates (MAPbI_3_:quartz). By contrasting photoluminescence (PL) intensities and transient decays of these samples against those of polymers deposited straight onto quartz, we are able to demonstrate clear evidence of ET occurring from photoexcited polymer films to the MAPbI_3_ layer. Based on these measurements, we create a model that captures the ET process and its efficiency in this geometry. First, we derive a mathematical formalism for Förster-type ET in restricted planar geometries between two layers of different materials under front photoexcitation. Using PL transient-decay measurements, we calibrate the global input parameters of the formulae and show that the mathematical formalism well describes our data. Second, we use these results to model the ET efficiencies as a function of different parameters, namely polymer film thickness, *L*, polymer PL quantum efficiency (PLQE), *ϕ*_d_ and the absorption coefficient of the polymer at the wavelength of the incoming light, *α*(*λ*_exc_). The model we present is thus generally applicable to determine the efficiency of ET between organic semiconductors and MHPs, based on easily accessible physical quantities. We find that for thin (≤10 nm) organic charge-extraction layers that exhibit high PLQE, much of the absorbed energy is transferred to the MHP layer, meaning that ET can be used as an effective tool to mitigate absorption losses in CTLs. To explore if the morphology of such thin polymer layers is affected by interface formation with an MHP, we further conduct a careful analysis of the PL spectra of P3HT (poly(3-hexylthiophene-2-5-diyl)) for a range of film thicknesses, highlighting subtle conformational changes.

## Results

### Experimental observation of energy transfer

**Fig. 1:**
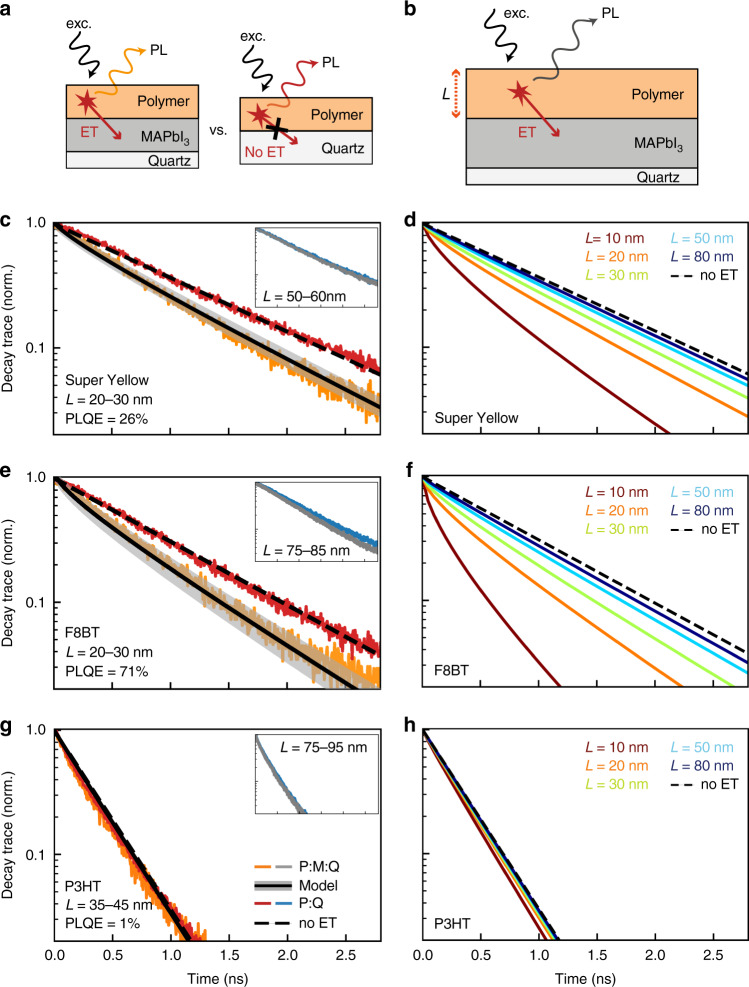
PL transients revealing energy transfer between polymer and MAPbI_3_ thin films. **a** Samples examined were either thin films of polymers deposited on MAPbI_3_ films on quartz (schematic diagram on the left) or polymers deposited directly on quartz to serve as references (schematic diagram on the right). ET may occur between the polymer and the MAPbI_3_ layer, leading to PL quenching, as depicted schematically in **b**. The left column shows time-resolved PL transients as a function of time after excitation for polymers **c** Super Yellow, **e** F8BT and **g** P3HT, when deposited as films of thickness *L* on either MAPbI_3_:quartz (orange lines) or just on quartz (red lines). The dashed lines are monoexponential fits to the data, yielding the PL lifetime of each polymer on quartz in the absence of ET (lifetime values provided in Supplementary Table 4). The solid black lines are fits to data based on an ET model described in the main text, with the grey shaded area indicating the error margin. The insets show equivalent data for thicker polymer films on either MAPbI_3_:quartz (grey lines) or just on quartz (blue lines). The right column depicts the expected PL-decay traces for polymers **d** Super Yellow, **f** F8BT and **h** P3HT for different film thickness *L*, as determined from the ET model. PL transients for the scarcely emitting PTAA samples are provided as Supplementary Fig. 15.

To explore excitation ET between a conjugated polymer layer and MAPbI_3_, we investigated four different polymers that have been commonly used in optoelectronic devices, namely Super Yellow (PDY-132, a poly(1,4-phenylenevinylene)-based light-emitting copolymer developed by Merck Chemicals), F8BT (poly(9,9-dioctylfluorene-alt-benzothiadiazole)), P3HT (poly(3-hexylthiophene-2,5-diyl)) and PTAA ((poly[bis(4-phenyl)(2,4,6-trimethylphenyl) amine])). Super Yellow and F8BT are commonly used in electroluminescence applications such as light-emitting diodes^43,44^ because they exhibit high PLQE, which also makes them suitable reference candidates for our investigation of ET phenomena. P3HT and PTAA are commonly included in MHP-based solar cells as hole-transport layers^8,45^ and are more weakly emissive. Supplementary Table 1 lists the relevant energy levels of the highest-occupied and lowest-unoccupied molecular orbitals of these polymers, in comparison with the relevant energy levels for a range of MHPs, as extracted from the literature. As schematically indicated in Fig. 1a, we produced two types of samples comprising either thin films of polymers deposited on MAPbI_3_ films on quartz (referred to as “polymer:MAPbI_3_:quartz” in the following), or polymers deposited directly on quartz to serve as references (termed “polymer:quartz”). Polymer films were fabricated through dynamic spin coating from solution, with film thickness control achieved through the adjustment of spin speed. MAPbI_3_ layers were fabricated by thermal co-evaporation that is known to yield relatively smooth MHP surfaces that benefit the deposition of conformal polymer thin films^46^. Further details on sample-fabrication procedures are provided in the “Methods” section below.

The ultimate aim of our investigation is to map the parameter space determining the efficiency of ET between a photoexcited polymer layer and the underlying MAPbI_3_. For this purpose, we begin by quantifying experimentally how much of the energy from incoming light undesirably absorbed by the polymer is transferred to the MAPbI_3_ layer underneath. In order for us to understand and model such ET processes subsequently, we also need to determine all relevant physical quantities for each sample. We measured film thicknesses (Supplementary Tables 2 and 3), absorption spectra (Supplementary Figs. 2–9), steady-state PL (Supplementary Figs. 8–12) and PL-decay transients (Fig. 1 and Supplementary Figs. 13–15) for all samples, and determined the PLQE of all polymer films on quartz (Table 1 and Supplementary Fig. 16). Experimental details for all measurements are provided in the “Methods” section below.

**Table 1:** Förster radius and energy-transfer efficiencies for a range of polymers on MAPbI_3_. For each polymer type (Super Yellow, F8BT, P3HT and PTAA) deposited on MAPbI_3_:quartz, the extracted Förster radius is provided, giving a measure of the distance over which ET is effective for each polymer:MAPbI_3_ material pair. The stated overall efficiency of ET is specific to the polymer film thickness and PLQE stated.

Polymer	PLQE (%)	Thickness (nm)	Förster radius (nm)	ET efficiency (%)
Super Yellow	26	23	8.5 ± 0.6	28
F8BT	71	25	10 ± 0.9	38
P3HT	1	40	4.9 ± 0.7	3
PTAA	0.5	35	4.4 ± 0.5	2

In order for us to assess ET transfer experimentally, we compared transient PL-decay dynamics following front excitation of polymer films deposited on quartz, with those on MAPbI_3_:quartz (see schematic in Fig. 1a). The left column of Fig. 1 depicts such measured PL-decay traces for the polymers Super Yellow, F8BT and P3HT, with data for PTAA provided in Supplementary Fig. 15. The samples were excited with laser pulses at wavelengths near the peak of the polymer absorption, and PL-decay traces were recorded at wavelengths at which predominantly the polymer emits (see Supplementary Table 4 for full details). We find that the measured PL transients of thin (~25 nm) films of the high-PLQE polymers Super Yellow and F8BT exhibit a faster decay when deposited on top of MAPbI_3_:quartz compared with the respective polymer:quartz reference samples. This observation implies that an additional decay channel exists for excitations in these samples, which is suppressed for thicker (>50 nm) polymer layers and the more weakly emitting polymers P3HT and PTAA. These findings are further supported by matching reductions in the overall PL emission intensities when a thin layer of highly emissive polymers Super Yellow and F8BT is deposited on MAPbI_3_:quartz, as opposed to just quartz. Such PL-intensity quenching is again suppressed for polymer-layer thickness greater than 50 nm or for weakly emitting polymers (see Supplementary Figs. 10–12). We note that only the observation of accelerated PL decays decisively indicates the presence of ET between the polymer layer and MAPbI_3_. The observed PL-intensity quenching, while compatible with such ET, could also be influenced by additional processes such as photon reabsorption.

Our observations of PL-intensity quenching and lifetime shortening for thin polymer films deposited on MAPbI_3_ clearly indicate that excitation energy is transferred from the polymer to MAPbI_3_. Faster PL decay for an energy donor material in the presence of an energy acceptor is a hallmark of resonant ET^27,47–49^, as is reduced PL intensity of the donor in the presence of an acceptor^24,27^. Photoexcitation of the polymer layer will generate relatively tightly bound excitons in the material^50^, whose dipole moments are associated with oscillating electric fields that will couple to electronic transitions in the perovskite. Such dipole–dipole resonant coupling may lead to transfer of the originally generated exciton from the energy donor (the polymer) to the acceptor (MAPbI_3_) without the need for photon emission, provided that the donor emission and acceptor absorption spectrally overlap. We note that an ET process that couples to states high above the band edge of MAPbI_3_ will most likely result in the generation of free electron–hole pairs, rather than tightly bound excitons, given the low (15 meV)^51^ exciton-binding energy for MAPbI_3_.

**Fig. 2:**
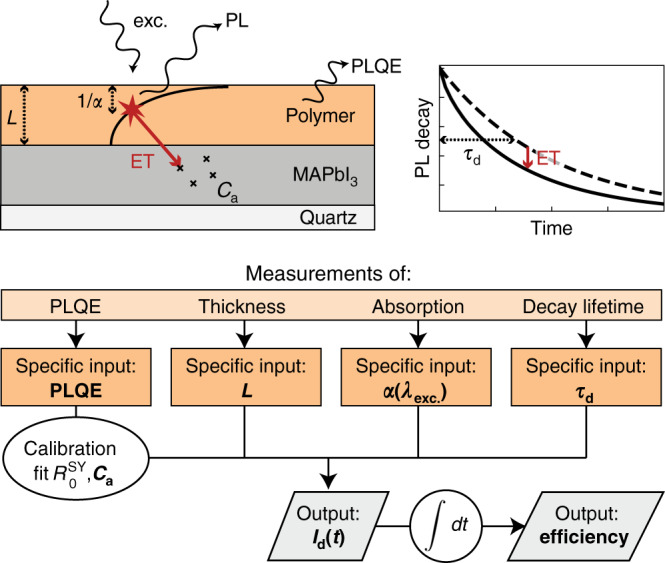
Schematic representation of the model describing ET in polymer:MAPbI_3_ two-layer films. The top right shows the two-layer sample architecture on quartz substrates and the typical excitation geometry. Polymer excitation from the front surface causes an exponentially decaying profile in the polymer layer. ET between the polymer and the underlying MAPbI_3_ results in accelerated PL decays (top right). The bottom graphic shows the hierarchy of the modelling and its dependence on input parameters: polymer film thickness *L*, PL lifetime *τ*_d_ of the polymer in the absence of MAPbI_3_, polymer PLQE and polymerabsorption coefficient at the wavelength of excitation *α*(*λ*_exc_). The Förster radius ${R}_{0}^{{\rm{SY}}}$ for ET between Super Yellow and MAPbI_3_, and the nominal acceptor concentration *C*_a_ that applies to MAPbI_3_, are first determined from fits to PL-decay transients for Super Yellow:MAPbI_3_ samples, and used to calibrate modelled transients for samples involving the other polymers.

In addition, this ET mechanism is indeed intuitively expected to be more prominent for thinner polymer films and higher PLQE values, as we observe here. Such dependence of the ET efficiency on PLQE is a characteristic feature of Förster-resonant ET, and would not be expected to occur for charge transfer^27^. For thinner films, a larger fraction of the photoexcited excitons is created in the polymer film within close proximity of the polymer:MAPbI_3_ interface. Given that the ET mechanism only has a certain range over which it is effective (quantified by the Förster radius^52^, discussed in more detail below), thinner polymer films therefore result in a higher likelihood of excitons being transferred to MAPbI_3_. This situation is further exacerbated when polymer layers are highly absorbing, since illumination creates an exponentially decaying excitation profile within the film (see schematic in Fig. 2), whose penetration depth depends inversely on the polymer-absorption coefficient^53^. For high absorption coefficients at given excitation wavelengths, excitons are thus generated close to the surface of the polymer film, far from the polymer:MAPbI_3_ interface, if films are thick. In addition, the observed strong dependence of the ET process on the PLQE of the polymer clearly supports the presence of ET, given that a lower PLQE may reflect a reduced oscillator strength of the excitonic downward transition in the polymer, which will lower the coupling strength to electronic transitions in MAPbI_3_. As discussed in more detail below, P3HT has a strong tendency for H-aggregate formation, which suppresses its PLQE and oscillator strength for downward transitions, explaining why even for thin P3HT films on MAPbI_3_ little ET occurs (see Fig. 1g).

Taken together, these considerations provide strong evidence for ET occurring between a thin layer of highly emissive polymer and an underlying MAPbI_3_ layer. To capture such processes quantitatively, we proceed by creating a model that accurately reproduces the experimentally measured PL decays. Based on this model, we are able to determine how properties of CTLs, such as PLQE and lifetimes, layer thickness and the absorption spectrum links with ET efficiency. This approach will provide design criteria for CTLs exhibiting efficient ET from the photoexcited CTL to the MHP, opening a pathway towards reduced parasitic absorption losses in CTLs and improved performance of perovskite-based solar cells.

### FRET in two-layer structures

A correct model of any ET process always has to take account of the layer geometry, which in our present case, comprises a two-layer structure photoexcited from the front surface (see schematic in Fig. 2). A variety of theoretical approaches have been applied to adapt Förster’s theory of resonant ET to consider the effect of varying spatial dimensions^28–32^. Yekta et al.^54^ derived analytic expressions for the PL decay of an energy donor in the presence of an energy acceptor for systems with planar symmetry, such as for the case of “parallel slabs of donor and acceptor touching at a sharp interface”, which applies to the situation of a polymer film deposited on a MAPbI_3_ layer. We adapted their approach to introduce a non-uniform donor concentration *C*_d_(*z*), accounting for the fact that the number of initially photoexcited donors as a function of film depth *z* is proportional to a Beer–Lambert profile^53^. We thus find the following expression for the donor decay *I*_d_(*t*):

1 $${I}_{{\rm{d}}}(t)\,=\,{e}^{-t/{\tau }_{{\rm{d}}}}\,{C}_{{\rm{d}}}\,\int_{0}^{L}{e}^{-\alpha (L-z)}\,\varphi (z,t)\,dz,$$

with

2 $$\varphi (z,t)\,=\,\exp \left(-\frac{\pi }{3}\,{C}_{{\rm{a}}}\,{R}_{0}^{3}\,{\left(\frac{t}{{\tau }_{{\rm{d}}}}\right)}^{\frac{1}{2}}\,[{T}^{-\frac{1}{2}}(1-{e}^{-T})+2\gamma (\frac{1}{2},T)-3{T}^{-\frac{1}{6}}\,\gamma (\frac{2}{3},T)]\right),$$

where the substitution

3 $$T\,=\,\frac{t}{{\tau }_{{\rm{d}}}}\,{\left(\frac{{R}_{0}}{z}\right)}^{6},$$

has been made and *γ*(*n*, *T*) is the incomplete gamma function, defined as

4 $$\gamma (n,T)\,=\,\mathop{\int}\nolimits_{0}^{T}\,{u}^{n-1}\,{e}^{-u}\,du.$$

*R*_0_ denotes the Förster radius, which is the characteristic distance between a donor and an acceptor at which any other pathway of donor de-excitation is as likely to occur as de-excitation via ET

5 $${R}_{0}\,=\,{\left(\frac{9\mathrm{ln}\,(10){\kappa }^{2}{\phi }_{{\rm{d}}}}{128{\pi }^{5}{N}_{{\rm{A}}}{n}^{4}}J\right)}^{1/6},$$

where *κ*^2^ accounts for the geometric orientation of the donor and acceptor dipoles (here, $\kappa =0.845\sqrt{2/3}$, assuming random but fixed relative orientations of donor and acceptor dipoles within a solid film^55^), *ϕ*_d_ is the PLQE of the polymer:quartz samples in the absence of MAPbI_3_, *N*_A_ denotes the Avogadro constant, *n* is the refractive index and *J* is the overlap integral between donor emission and acceptor absorption^24,27^. The other parameters in Eqs. (1)–(3) are the donor (polymer) lifetime *τ*_d_, donor (polymer) and acceptor (MAPbI_3_) concentrations, *C*_d_ and *C*_a_ respectively and the polymer film thickness *L* and absorption coefficient *α* at the wavelength of photoexcitation, *λ*_exc_. Eqs. (1)–(4) thus provide a framework for modelling the PL-decay data of the polymers on MAPbI_3_:quartz. An in-depth discussion of the approach is provided in Supplementary Note 1, which includes a full analytical derivation as well as discussion of parameter choices and modelling.

### PL-decay modelling

Modelling of the PL-decay data was performed in two steps, based on the expression for *I*_d_(*t*) in Eq. (1). As the first step, we modelled the data set associated with the polymer Super Yellow, because it showed sufficiently strong ET effects. From these fits to data, we determined a value for the nominal acceptor concentration *C*_a_ that applies to MAPbI_3_ and is therefore set to remain unchanged from here onwards. We further determined the associated Förster radius ${R}_{0}^{{\rm{SY}}}$ for ET between Super Yellow and MAPbI_3_. In the second step, we model the PL transients for the remaining polymer:MAPbI_3_ samples, assuming that any differences in *R*_0_ between the polymers result from changes in the PLQE of polymer, *ϕ*_d_, only (see Eq. (5), and Eq. S26 in Supplementary Note 1). We then use the measured PLQE values to calculate the expected PL transients *I*_d_(*t*) for the polymers F8BT, P3HT and PTAA on MAPbI_3_ through Eq. (1). A schematic overview of the modelling process is depicted in Fig. 2, and a detailed assessment of the input parameters is outlined in Supplementary Note 1. Our modelling reveals that there is a limited set of CTL properties that are decisive to the ET process. These polymer-specific input parameters are polymer film thickness *L*, PL lifetime *τ*_d_ of the polymer in the absence of MAPbI_3_, polymer PLQE *ϕ*_d_ and polymer-absorption coefficient at the wavelength of excitation *α*(*λ*_exc_). Having determined the cornerstones of the ET process, we are thus in a position to calculate the efficiency of the ET for a range of different scenarios, as detailed further below.

We find excellent agreement between the measured and modelled PL-decay traces, which confirms our methodological approach. Figure 1 shows modelled PL-decay traces for polymer:MAPbI_3_:quartz samples as black lines superimposed on the experimental data (orange) in the left column, with errors (grey shaded areas) deduced from measurement uncertainties. We are also able to extrapolate from such models how PL-decay traces would be expected to change for different polymer-layer thicknesses *L*, as shown in the right column of Fig. 1. Trends are in agreement with what would intuitively be expected, i.e., the modelled PL transients decay faster for thinner polymer films on MAPbI_3_:quartz because ET is more efficient if excitations are created in these polymer films closer to the interface with MAPbI_3_. In addition, the experimental and modelled trends confirm that a high polymer PLQE *ϕ*_d_ enlarges the dipole–dipole coupling between donor and acceptor, represented by the Förster radius *R*_0_ (c.f. Eq. (5)). Both experimental and modelled PL transients of polymer:MAPbI_3_:quartz samples show most clearly accelerated decays for F8BT, which has the highest PLQE (see Table 1) and indiscernible effects for the low-PLQE polymers P3HT and PTAA.

We further examine the magnitude of the Förster radius, which is a distance measure for ET and thus provides the first benchmark on the CTL thickness required to achieve efficient ET. *R*_0_ values were determined for each polymer:MAPbI_3_ pair as part of the modelling process and are listed in Table 1. The observed variations in *R*_0_ reflect the changes in PLQE of the polymer layers, given that ${R}_{0} \sim {\phi }_{{\rm{d}}}^{1/6}$ (Eq. (5)). Thus, for the most emissive polymers Super Yellow and F8BT, a Förster radius in the range of 8.5–10 nm is determined, but this value drops to 4.4–4.9 nm for the weakly emitting PTAA and P3HT. These trends clearly demonstrate that the more weakly emissive the CTL material is, the thinner this layer must be in order for ET to remain effective. However, we note that *R*_0_ values are relatively high for all polymers, which results from the complete spectral overlap between the polymer emission and MAPbI_3_ absorption (c.f. Supplementary Figs. 8–12), thereby fulfilling the resonance condition for ET over a wide range of wavelengths^52^. This scenario will generally apply to single-junction solar cells based on MHP materials, because they absorb through the full visible range, typically up to  ~1.6 eV, while any CTL emission will fall into the UV–visible range by design. Therefore, such ET processes have great potential to ameliorate parasitic light-absorption losses in the CTL. In addition to such resonance effects, exciton diffusion within the donor material prior to the ET event has been observed to enhance the Förster radius deduced from PL-decay dynamics^47,56,57^, and may also be at play here.

### Assessing the efficiency of the energy transfer

Our model allows us to develop design criteria for efficient ET, by assessing how CTL properties such as layer thickness, PLQE and absorption coefficient must be optimised. Such an approach is generally applicable to semiconducting CTLs deposited on MAPbI_3_, depending only on easily accessible material parameters. Here we focus in particular on calculating the absolute ET efficiency for a particular parameter space, which is the fraction of excitons created in the CTL layer that subsequently undertakes ET to the underlying MAPbI_3_. Such efficiency calculations enable a clear assessment of the extent to which such parasitic light-absorption losses in CTLs can be mitigated by ET, and are therefore the best metric to feed into any device-efficiency analysis and modelling. We first determine ET efficiencies for the specific cases of polymers Super Yellow, F8BT, P3HT and PTAA on MAPbI_3_, and then generalise the results for CTLs as a function of their properties.

We may relate the ET efficiency to the decay of photoexcitation inside a CTL material in the absence and presence of the MAPbI_3_ film. By integrating the PL-decay transients over time after excitation, we obtain a measure of the residual population decay in either case. While in the absence of MAPbI_3_, the time integral scales with the total density of excitations generated in the CTL; in the presence of MAPbI_3_, it only yields the number density for which ET has failed to occur. Therefore, the difference in the time integral over the appropriately scaled monoexponential PL decay in the absence of MAPbI_3_ and the time integral over the donor (CTL) PL decay represented by Eq. (1) determines the transfer efficiency *η*_ET_, as follows:

6 $${\eta }_{{\rm{ET}}}=\frac{{F}_{{\rm{sc}}}\,\int\exp (-t/{t}_{{\rm{d}}})\,dt-\int{I}_{{\rm{d}}}(t)\,dt}{{F}_{{\rm{sc}}}\,\int\exp (-t/{t}_{{\rm{d}}})\,dt}.$$

Here, the scaling factor *F*_sc_ = *I*_d_(*t*_0_) was introduced to normalise the PL decays for the CTL in the presence and absence of MAPbI_3_ at the time *t*_0_ of excitation, by which no significant ET had yet occurred. Input parameters specific to the CTL are its film thickness *L*, PL lifetime *τ*_d_, PLQE *ϕ*_d_ and the absorption coefficient *α*(*λ*_exc_) at the wavelength of excitation. Full details on the calculation of the ET efficiency are provided in Supplementary Note 1.3. We note that while Eq. (6) is formulated in terms of pulsed photoexcitation, the calculated efficiency values will also hold under continuous-wave illumination, given that photophysical processes in such CTLs are typically excitonic and monomolecular for excitation densities that easily encompass typical solar illumination conditions.

Using the curves and parameters determined earlier when modelling ET for our specific polymer:MAPbI_3_ samples, we proceed to calculate the ET transfer efficiency for each combination, based on Eq. (6), with the resulting values listed in Table 1. For the high-PLQE polymers Super Yellow and F8BT, good ET efficiencies of 28–38% are determined for ~25-nm layers, highlighting excellent potential of these mechanisms for reducing parasitic light-absorption losses. However, for P3HT and PTAA, the combination of low-PLQE values (≤1%) and somewhat larger layer thicknesses results in low ET efficiencies of only 2–3%. As our further analysis indicates below, all of these ET efficiency values could be boosted significantly if thinner polymer films were employed.

**Fig. 3:**
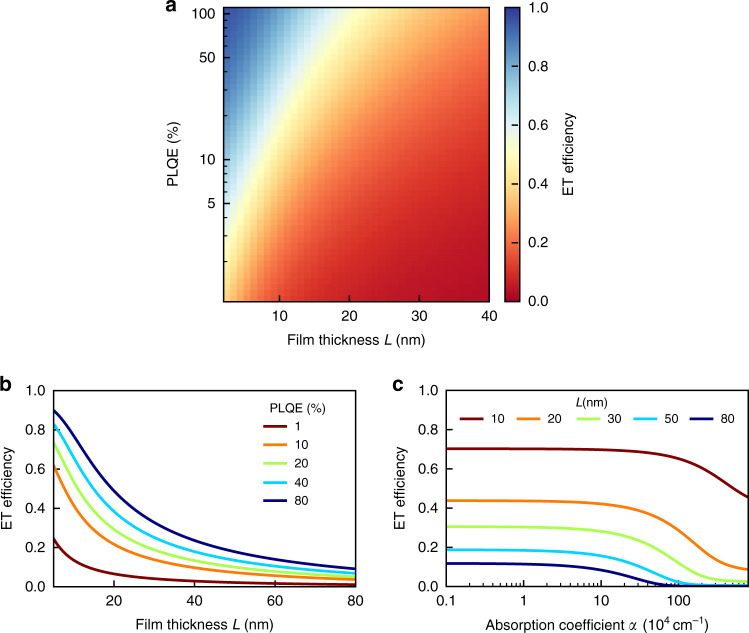
Modelled ET efficiency between a semiconducting charge-transport layer and a MAPbI_3_ layer. ET efficiency as a function of the PLQE, film thickness and absorption coefficient of the CTL. **a** False-colour plot indicating the efficiency of ET between a photoexcited CTL and a MAPbI_3_ layer underneath, as a function of organic CTL thickness *L* and PLQE. **b** ET efficiency as a function of semiconductor CTL thickness *L* for distinct PLQE values, and absorption coefficient *α*(*λ*_exc_) = 13 × 10^4^ cm^−1^. **c** ET efficiency as a function of the CTL absorption coefficient *α*(*λ*_exc_) at the excitation wavelength, for different film thicknesses and a value of 50% PLQE. Additional false-colour plots mapping out this parameter space with wider ranges are shown in Supplementary Fig. 17.

By modelling the ET efficiency more generally as a function of CTL film thickness, PLQE and absorption coefficient at the excitation wavelength, we are able to develop design criteria for mitigating parasitic light absorption through ET. Figure 3 illustrates in a number of different graphs how the ET efficiency varies with these parameters. We find three key trends that indicate how efficient ET from front-facing CTL to an underlying MAPbI_3_ layer can be achieved: (i) the higher the PLQE of the CTL, the more efficient the ET. (ii) Thin (*L* ≤ 10 nm) CTLs are beneficial for efficient ET. (iii) A high (≫1 × 10^5^ cm^−1^) absorption coefficient of the organic semiconductor at the wavelength of the absorbed light impedes ET. The underlying reasons for these observations relate to both the nature of resonant ET and the dual-layer geometry. As elucidated previously, high PLQE leads to stronger dipole–dipole coupling between downward transitions in the photoexcited CTL and upward transitions responsible for absorption in MAPbI_3_. CTLs whose thickness is comparable with the Förster radius *R*_0_ that captures the range over which dipole–dipole interactions are effective, will also show enhanced chance of ET events. A high absorption coefficient *α*(*λ*_exc_) at the excitation wavelength, on the other hand, is detrimental, because it results in a short penetration depth of incoming light, with more photoexcitations being created in the CTL closer to its front surface and therefore further away from the MAPbI_3_ layer, reducing ET efficiency. Such uneven excitation profiles will become prominent when *α*(*λ*_exc_) ≫ *L*^−1^, which, for CTL-layer thicknesses of a few tens of nanometres, means a threshold condition of *α*(*λ*_exc_) ~10^5^ cm^−1^ above which ET efficiency deteriorates, as shown in Fig. 3c (and Supplementary Fig. 17). For smaller values of *α*(*λ*_exc_), the penetration depth is longer than the layer thickness *L*, and more excitations are likely to be created within the capture radius of the MAPbI_3_ acceptor layer. We note that commonly employed CTL materials are likely to meet this criterion as they are generally chosen to have minimised absorption of sunlight^58^. We further found that the PL lifetime *τ*_d_ does not substantially influence the ET efficiency, within the wide range of lifetimes (0.1–1000 ns) and PLQE values (0.1–80%) we modelled.

### Morphology of P3HT thin films at the perovskite interface

As a final part of our investigation, we explore whether changes in the thickness of a polymer CTL might influence film morphology and thereby the electronic configuration of the material, which could in turn affect device performance. In addition, the precise nature of the interface formed between a polymer CTL and an MHP may influence chain conformation near such an interface. These effects may be particularly prominent for CTLs designed for high ET efficiencies to the underlying MHP, given that our previous examination of these processes suggested that they require very thin CTLs. P3HT offers an ideal system to study in this context, as this polymer is known to exhibit large spectral changes with differences in chain arrangement^41,59–65^.

**Fig. 4:**
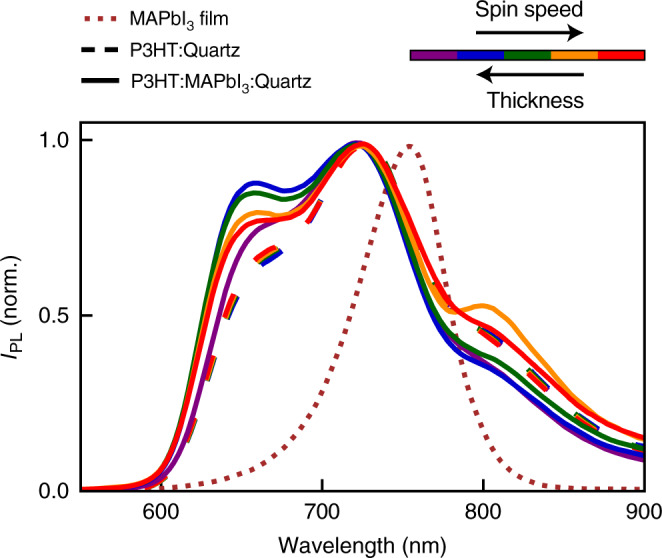
Changes in PL spectral shape when P3HT films of different thicknesses are deposited on MAPbI_3_ or quartz. Normalised PL intensity from P3HT films deposited through spin coating from solution with varying spin speeds onto either quartz (dashed lines) or MAPbI_3_:quartz (solid lines) substrates. The line colours indicate spin speeds that anti-correlate with the P3HT film thicknesses achieved. Spin speed was increased from 2000 to 6000 rpm in steps of 1000 rpm, yielding P3HT film thicknesses from 92 to 40 nm for the films on MAPbI_3_:quartz and 77 to 42 nm for the P3HT films on quartz (film thickness does not scale linearly with spin speed; the full list of values is provided in Supplementary Table 3). In addition, the normalised PL spectrum of a MAPbI_3_ film on quartz is shown as a dotted brown line.

In order to determine any dependencies of P3HT chain conformation on film thickness and interfaces, we examine PL spectra for P3HT films deposited either directly on quartz, or on MAPbI_3_:quartz substrates, as shown in Fig. 4. A variation in film thickness was achieved by tuning the spin speed, with faster spin speed resulting in thinner polymer films. We find that for deposition of P3HT films on MAPbI_3_:quartz (solid lines), significant modification of the vibronic peak structure occurs when the film thickness is altered. P3HT PL spectra in Fig. 4 display vibronic peaks at 655 nm (0–0), 725 nm (0–1) and 810 nm (0–2) (spectra are normalised to the 0–1 transition), whose relative amplitudes clearly change as film thickness is decreased from 92 to 40 nm. Such spectral transformations have been widely researched for P3HT^41,59–65^, and are well understood to arise from morphological changes associated with polymer aggregation through inter- (through space) and intrachain (through polymer bond) interactions (for a more in-depth discussion see Supplementary Note 2). We note that if P3HT films are instead deposited directly on quartz, variations in film thickness have little effect, although all of these films show a reduced ratio of the 0–0:0–1 peaks compared to films on MAPbI_3_:quartz (the very slight shifts near the high-energy end of the 0–0 feature around 650 nm are likely caused by self-absorption). We therefore propose that the interactions at the polymer:MAPbI_3_ interface have a significant effect on the resulting polymer chain morphology.

## Discussion

Overall, we highlight that it is the combination of CTL thickness, PLQE and absorption coefficient that is decisive for determining the attainable efficiency of ET. In particular, stronger parasitic light-absorption coefficients may still be tolerated for CTLs, if these layers are thin and exhibit high PLQE. This finding is important, given that exploration of sun-facing front CTL materials so far has concentrated exclusively on materials that are transparent (i.e., absorb as little as possible across the sun spectrum) to avoid parasitic absorption in the CTL^58^. Our findings suggest that less transparent materials with high PLQE may be equally tolerable as CTL materials for MHP-based solar cells because absorbed energy is efficiently transferred to the MHP. This approach opens up a larger material space suitable for CTLs, allowing selection of previously unexplored candidates that share further positive characteristics, such as higher charge-carrier mobilities or more optimised energy-level alignment, which can reduce charge-extraction losses. Regardless of the specific selection, any efficient ET process between the front-facing CTL and the active MHP layer will enhance device performance, as it mitigates parasitic light absorption by transferring excitations to the MHP, from where they can be harvested as photocurrent.

We discuss the observed spectral changes of the P3HT films at the perovskite interface in terms of the well-understood framework of inter- and intrachain interaction strengths. The observed trends upon switching substrate for P3HT deposition from quartz to MAPbI_3_:quartz, i.e., the blueshift and an increase in 0–0–0–1 PL peak ratio, correspond to a decrease in interchain coupling (weaker H-aggregate signature)^41,66^. In addition, comparing the P3HT:MAPbI_3_:quartz films of different thicknesses/spin speed, interchain coupling strengthens (the 0–0–0–1 PL ratio decreases) and intrachain coupling weakens (the 0–2–0–1 PL ratio increases) for the two thinnest films spun at thr highest spin speeds (solid red and yellow lines in Fig. 4)^66,67^.

Such changes in interaction strengths can be explained by differences in film morphology, resulting from variations in interface interactions and spin speeds. For example, weaker interchain (through-space) interactions and stronger intrachain (through-bond) interactions have been observed when P3HT forms a two-phase morphology of interconnected crystalline and amorphous phases, compared with films comprising a chain-extended morphology of layered stacks exhibiting high torsional disorder^66,67^. The evolution of the PL lineshape we observe for P3HT films on MAPbI_3_:quartz thus most likely derives from the presence of an amorphous phase formed under some conditions near the interface to MAPbI_3_, which causes a decrease in interchain interaction compared to the films deposited on a smoother quartz substrate^42,68^. As Ehrenreich et al.^42^ pointed out, such amorphous polymer structure may energetically enhance the HOMO (highest-occupied molecular orbital) level near the interface^69^, which can affect charge transport and injection^70^. We observe here that such an amorphous phase seems less prominent for the thinnest polymer films (~40 nm) on MAPbI_3_, which are produced at higher spin speeds. This effect could potentially derive from a higher spin speed causing more torsional disorder in the films, in particular when a substrate less smooth than quartz is used. Overall, these observations suggest that ultra-thin polymer films of desired chain morphology should be readily available through careful tuning of processing parameters. In addition, when aiming for ultra-thin polymer CTLs exhibiting efficient ET from the CTL to a MHP layer, the specific energetics of such polymer layers will have to be considered when favourable-level alignment across the interface is pursued.

In conclusion, we have demonstrated that efficient energy transfer may occur between a photoexcited organic semiconductor and a MHP layer. This mechanism is shown to have excellent potential for mitigating parasitic absorption of light that occurs in sun-facing charge-transport layers incorporated in perovskite solar cells. Significant energy transfer will reduce photocurrent losses in these devices, allowing them to approach their intrinsic limits. By modelling the experimentally observed energy-transfer process between a range of photoexcited polymer CTLs and an underlying MAPbI_3_ layer, we showed that high transfer efficiencies can be achieved for CTLs that are very thin (≤10 nm) and/or exhibit high PLQE. Importantly, semiconducting CTLs fulfilling these characteristics do not need to be fully transparent at the wavelengths of MHP absorption, which considerably widens the field of possible candidates for charge-extraction layers. Finally, we showed that when thin layers of P3HT are deposited on MAPbI_3_, spin speed during the fabrication and the resulting film thickness influence chain conformation and aggregation, which may in turn affect electronic energy levels. The deposition of ultra-thin CTLs on MHPs exhibiting optimised ET will therefore need to be tuned to take full account of any interaction prevalent at the CTL:MHP interface. Overall, our discovery that efficient ET can compensate for parasitic absorption losses in sun-facing CTLs will allow for a much wider range of organic CTLs to be explored in future perovskite solar cell architectures.

## Methods

### Sample preparation

*Substrate cleaning*: The z-cut quartz substrates were cleaned with Hellmanex solution followed by a thorough rinse with deionised water. The substrates were then washed with acetone, isopropanol and ethanol. In the last cleaning step, the substrates were plasma-etched in O_2_ for 10 min.

*Polymer films*: These were prepared by dynamic spin coating from solution. For each sample, 40 μL of the polymer solution were deposited on pre-cleaned quartz substrates for the polymer-only films and on evaporated MAPbI_3_ for the polymer:MAPbI_3_ films. The spin speed was varied for each polymer film deposition in order to achieve a difference in thickness (see further details for each polymer below), while the duration of the spinning process was kept at 45 s for all samples. All steps apart from the cleaning of the substrates were carried out under nitrogen atmosphere. The films were kept in a nitrogen glovebox before and in-between measurements.

*F8BT*: (Poly(9,9-dioctylfluorene-alt-benzothiadiazole)) with an average molecular weight of *M*_w_ > 20,000 (CAS number 210347-52-7) was purchased from Sigma-Aldrich. The “thick” F8BT films used for this study were spin-coated from a solution of F8BT in anhydrous toluene with a concentration of 10 mg mL^−1^ at a spin speed of 2000 rpm, the “thin” films from a solution with a concentration of 5 mg mL^−1^ of F8BT in anhydrous toluene at 5000 rpm.

*Super Yellow*: (Merck poly(1,4-phenylenevinylene)-based copolymer) with *M*_w_ ≥ 1,300,000  (CAS number 26009-24-5) was purchased from Sigma-Aldrich. The Super Yellow films were spin-coated from a solution of 5 mg mL^−1^ of the polymer in anhydrous toluene, the “thick” films at a spin speed of 1000 rpm and the “thin” films at 3000 rpm. An additional film was spin-coated at 4000 rpm, which was of comparable thickness to that of the films spin-coated at 3000 rpm, and all measurements from this film were in excellent agreement with the data collected for the 3000-rpm film sample.

*P3HT*: (Poly(3-hexylthiophene-2-5-diyl)) was purchased from Sigma-Aldrich (CAS number 104934-50-1) and dissolved in anhydrous toluene at a concentration of 15 mg mL^−1^. The solution was then deposited onto either quartz substrates, or onto the MAPbI_3_ thin film on quartz by dynamic spin coating with rotation speeds of 2000, 3000, 4000, 5000 and 6000 rpm, to vary the thickness (see Supplementary Table 3) of the P3HT layers. For films termed “thick” and “thin”, the spin speeds 2000 rpm and 6000 rpm were used. The films were prepared on a thin MAPbI_3_ (65-nm) layer on a z-cut quartz substrate in order to allow for the separation of absorption components arising from the polymer and the MAPbI_3_. In addition, two control samples of different P3HT thickness were prepared on a 260-nm MAPbI_3_ film, showing the same photophysical behaviour, compared to those of the films on the thinner MAPbI_3_ layers on quartz.

*PTAA*: (Poly[bis(4-phenyl)(2,4,6-trimethylphenyl)amine]) was purchased from LUMTEC (CAS number 1333317-99-9) and dissolved in anhydrous toluene at a concentration of 15 mg mL^−1^. The solution was then deposited by dynamic spin coating with different spin speeds. The spin speeds used were 2000 and 6000 rpm for the “thick” and “thin” films, respectively.

*MAPbI*_*3*_: (Methylammonium lead iodide) layers were fabricated using thermal evaporation^71^. In brief, MAI and PbI_2_ were placed in separate crucibles, and the substrates were mounted on a rotating substrate holder to ensure that a uniform film was deposited. The temperature of the substrates was kept at 21 °C throughout the deposition. The chamber was evacuated to reach a high vacuum (~10^−6^ mbar), before heating of the PbI_2_ and the MAI. The substrates were then exposed to the vapour with an average deposition rate of 0.4 Å s^−1^. The rates of both the MAI and PbI_2_ deposition were monitored using quartz-crystal microbalances. The thickness of the MAPbI_3_ thin film was set by controlling the exposure time of the substrates to MAPbI_3_ precursor vapour. Three film thicknesses of MAPbI_3_ were used in this work: 450 nm (F8BT and Super Yellow samples), 260 nm (PTAA and P3HT control samples) and 65 nm (P3HT samples). Since all of these thicknesses significantly exceed the Förster radii determined for ET in polymer:MAPbI_3_ dual layers, the thickness of the MAPbI_3_ layer has no effect on the ET dynamics or efficiency.

*Film thicknesses*: These were measured using a Veeco 150 Dektak profilometer and were cross-checked with the respective absorption data. All samples were scratched with a sharp razor blade down to the z-cut quartz substrate, and the total thickness of each sample was determined. For the polymer:MAPbI_3_:quartz samples, the known thickness of the MAPbI_3_ layer was subtracted to obtain the polymer film thickness. The relative error in the determined film thickness value is estimated from repeated Dektak measurements on different spots on the films to be of the order of 10%. Full listing of values of the thicknesses of all polymer films fabricated for different substrates and spin speeds is provided in Supplementary Tables 2 and 3.

### Optical measurements

*Absorption measurements*: Reflection and transmission of samples were measured with a Bruker Vertex 80v Fourier-Transform Infrared (FTIR) spectrometer. As light sources, a tungsten halogen lamp (NIR source) and a deuterium lamp were used, and different spectral ranges were detected with a gallium phosphide (GaP) and a silicon (Si) diode detector. Absorption spectra were calculated from background light-corrected reflection (*R*) and transmission (*T*) as $-\mathrm{ln}\,(\frac{T}{1-R})$ and the spectra were baseline-corrected for scattering. The absorption spectra for all samples investigated are provided in Supplementary Figs. 2–9 and more details are given in Supplementary Methods for P3HT.

*Steady-state PL spectra*: The samples were excited with a mode-locked Ti:Sapphire laser at a repetition rate of 80 MHz and a pulse duration of 80 fs for all PL measurements (steady-state spectra, transients and PLQE). PL spectra were recorded using a Jobin Yvon Triax 190 monochromator and a nitrogen-cooled charge- coupled device Jobin Yvon Symphony silicon detector. All PL spectra are corrected for spectral response with a known lamp spectrum, also accounting for any filter used. Details of the excitation specific to each polymer are given in the Supplementary Methods. PL spectra for all samples are shown as Supplementary Figs. 8–12.

*PL-decay measurements*: The PL decays following pulsed photoexcitation were measured by time-correlated single-photon counting (TCSPC) using a PicoQuant PDM Series single-photon avalanche diode detector, which allows for a time resolution of 40 ps^72^. The samples were kept under vacuum during the experiment. Supplementary Table 4 provides an overview of excitation and detection wavelengths used for each polymer type, together with the absorption coefficient *α* at the excitation wavelength. In addition, decay lifetimes for the different polymers on quartz are listed, as obtained from monoexponential fits to the data in the time ranges 0.1–3 ns (F8BT and Super Yellow) and 0.1–1.5 ns (P3HT and PTAA). PL transients for all samples are shown in Fig. 1 and Supplementary Figs. 13–15.

*Photoluminescence quantum efficiency:* PLQE was determined for all polymer films on quartz by comparison of the integrated PL intensities with that for a sample of known PLQE standard, taking into account the difference in absorption at the excitation wavelength, as described in detail in Supplementary Methods.

## Supplementary information

Supplementary Information

## Data Availability

The data that support the findings of this study are available from the corresponding author upon reasonable request.
