# Supplementary material for: Efficient energy transfer mitigates parasitic light absorption in molecular charge-extraction layers for perovskite solar cells
Source: Nat Commun. 2020 Nov 2;11:5525. doi: 10.1038/s41467-020-19268-w (PMC7606526; doi:10.1038/s41467-020-19268-w)
Supplement: Supplementary file 1 — Supplementary Information [file 41467_2020_19268_MOESM1_ESM.pdf]

Supplementary Information for:

Efficient Energy Transfer Mitigates Parasitic Light Absorption in Molecular Charge-Extraction Layers for Perovskite Solar Cells

Hannah J. Eggimann, Jay B. Patel, Michael B. Johnston and Laura M. Herz

Supplementary Methods

1 Details of Absorption Measurements

The absorption spectra of the different polymers are depicted as Supplementary Figures below. Spectra were recorded using a range of different source/detector combinations (sources: tungsten halogen lamp (NIR source) and deuterium lamp, detectors: GaP and Si diode detectors) in order to cover the full range from the low-wavelength absorption of the polymers to the higher-wavelength absorption edge of $MAPbI_3$. For most polymer:$MAPbI_3$:quartz samples, the $MAPbI_3$ absorption is the dominant feature in the spectra and the specific polymer absorption cannot be extracted. However, for the P3HT samples on $MAPbI_3$ a much thinner (65 nm) $MAPbI_3$ layer was used to enable extraction of the polymer absorption by subtracting the absorption spectrum of a $MAPbI_3$:quartz film from the absorption spectra of the P3HT:$MAPbI_3$:quartz films (see Fig. S4). The resulting P3HT absorption spectra for films of different thicknesses, and the $MAPbI_3$:quartz absorption spectrum used for the subtraction, are shown in Fig. S5. This approach enables us to investigate polymer aggregation effects in P3HT films deposited on a $MAPbI_3$ layer by comparing the P3HT-component of the absorption spectrum for P3HT films of different thickness and spin speed on the quartz:$MAPbI_3$ substrates. Table S2 lists the thicknesses of all polymer films investigated in this study.

2 Details of PL Measurements

In addition to the description of PL measurement details provided in the Methods section of the main text, we list here polymer-specific excitation wavelengths and fluences for the steady-state PL spectra, and the detection wavelengths for the PL transient measurements. Table S4 provides for the different polymers on quartz an overview of the excitation and detection wavelengths, and decay lifetimes obtained from mono-exponential fits to the data in the time ranges 0.1–3 ns (F8BT and Super Yellow) and 0.1–1.5 ns (P3HT and PTAA).

F8BT

The PL excitation decays of F8BT films of different thickness on quartz and on $MAPbI_3$:quartz substrates are shown in Fig. 1 in the main text. The samples were excited at a wavelength of 400 nm. The PL excitation decay was observed at 540 nm. No fluence dependence was observed and the excitation fluence for the decays shown was $3\,nJ\,cm^{-2}$.

Super Yellow

The PL excitation decays of Super Yellow films of different thickness on quartz and on $MAPbI_3$:quartz substrates are shown in Fig. 1 in the main text. The samples were excited at a wavelength of 400 nm. The PL excitation decay was observed at 600 nm. No fluence dependence was observed and the excitation fluence for the decays shown was $3\,nJ\,cm^{-2}$.

P3HT

The P3HT films were excited at 460 nm with a fluence of 3–4 $nJ\,cm^{-2}$ and the decay traces were detected at 725 nm. The excitation decays of the thickest and thinnest film are shown in Fig. 1 in the main text. A full overview of TCPSC traces recorded for P3HT films of varying thicknesses on quartz and on $MAPbI_3$:quartz is shown in Fig. S13.

PTAA

The excitation wavelength for PL, TCSPC and PLQE was 360 nm with a fluence of the order of $1\,nJ\,cm^{-2}$; the detection wavelength for TCSPC was at 460 nm. The TCSPC traces of PTAA films of different thickness (between 20 nm and 100 nm on quartz and on $MAPbI_3$:quartz substrates (see Table S2 for the individual samples) are shown in Fig. S15 a). Additionally, the modelled decay traces are shown in Fig. S15 b). The decay lifetime was obtained from a mono-exponential fit to the TCSPC data from 0.1 ns to 1.5 ns.

3 Details of Photoluminescence Quantum Efficiency Measurements

Photoluminescence Quantum Efficiency (PLQE) was measured for all polymer films on quartz. PLQE, ϕ, can be obtained by comparison of the integrated PL intensity from the sample with a known PLQE standard with ϕ_{ref}, taking into account the difference in absorption at the excitation wavelength λ_{exc} [1]:

$$\phi_{\text{sample}} = \phi_{\text{ref}} \frac{I_{\text{sample}}(\lambda_{\text{PL}})}{I_{\text{ref}}(\lambda_{\text{PL}})} \frac{A_{\text{ref}}(\lambda_{\text{exc}})}{A_{\text{sample}}(\lambda_{\text{exc}})}, \tag{S.1}$$

where $I_{\text{sample/ref}}(\lambda_{\text{PL}})$ is the integrated PL intensity over the full spectral range of polymer/ reference emission and $A_{\text{sample/ref}}(\lambda_{\text{exc}})$ is the absorbance at the excitation wavelength for the PL of the polymer or reference, respectively. The reference standard used in this work is the well-known fluorescence standard Rhodamin 6G (Rhodamine 6G analytic standard from Sigma Aldrich, molar weight: $479\,\text{g}\,\text{mol}^{-1}$, $\phi_{\text{ref}} = 15$), which was diluted in solution with ethanol at a concentration of 0.8 mM. The suitability of this concentration was tested in previous work [2]. PL and Absorbance (O.D.) of the Rhodamine 6G solution are shown in Fig. S16. The polymer films degrade under simultaneous exposure to ambient air and laser excitation, therefore the films were measured in a vacuum cell. Because little light scattering is expected to occur for these films, the use of the vacuum cell instead of an integrating sphere should not lead to substantial errors. Additionally, for the energy transfer modelling, the comparison between data and model results is only sensitive to the relative PLQE values, making this approach particularly suitable here. The PLQE of Super Yellow was obtained by referencing with the fluorescence standard Rhodamine 6G in dilute solution in a quartz cuvette and was then used as a reference for the other polymers. Special care was taken for adjusting the excitation focus position for each film. The resulting PLQE values are listed in Table S4. The error given indicates the range of values obtained from a range of measurements for each polymer from thick and thin polymer films referenced either to the Rhodamine 6G solution (Super Yellow) or to the thick and thin Super Yellow samples (all other polymers).

Supplementary Figures

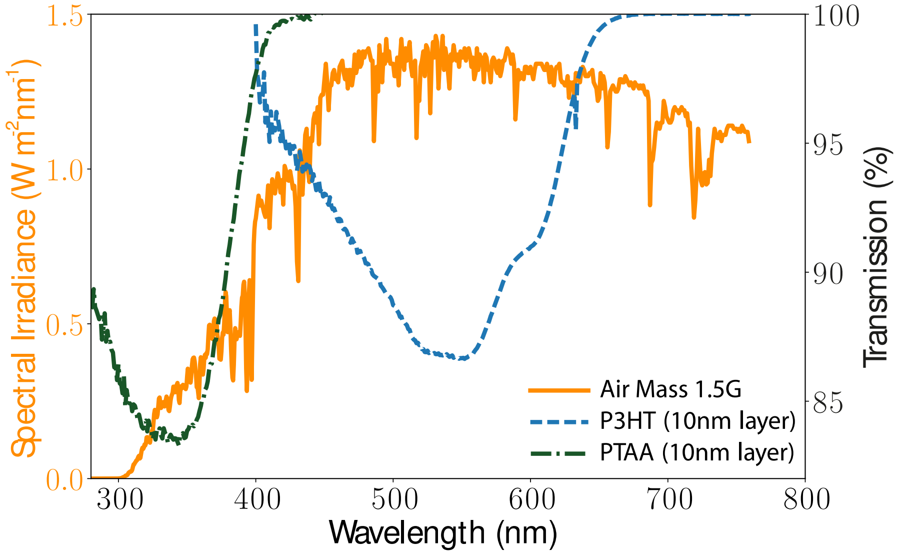

Supplementary Figure 1: Parasitic absorption of the solar spectrum from thin layers of PTAA and P3HT. The solar spectrum (orange) is taken from NREL [3] and the polymer transmission (blue dashed line for a 10 nm layer of P3HT and greeen dash-dotted line for a 10 nm layer of PTAA) is calculated from absorption data of each polymer. The relative losses incurred from parasitic absorption in the sun-facing polymer layer is calculated by integration over the polymer-layer filtered sun-light and division by the integral over the unfiltered light, within the spectral region up to 760 nm (the band edge of $MAPbI_3$). For a 10 nm polymer layer, 1% of the solar spectrum is lost for PTAA and 6% are lost for P3HT, which increases to 11% for a 20 nm-thick layer of P3HT.

Absorption Spectra of Polymer Films

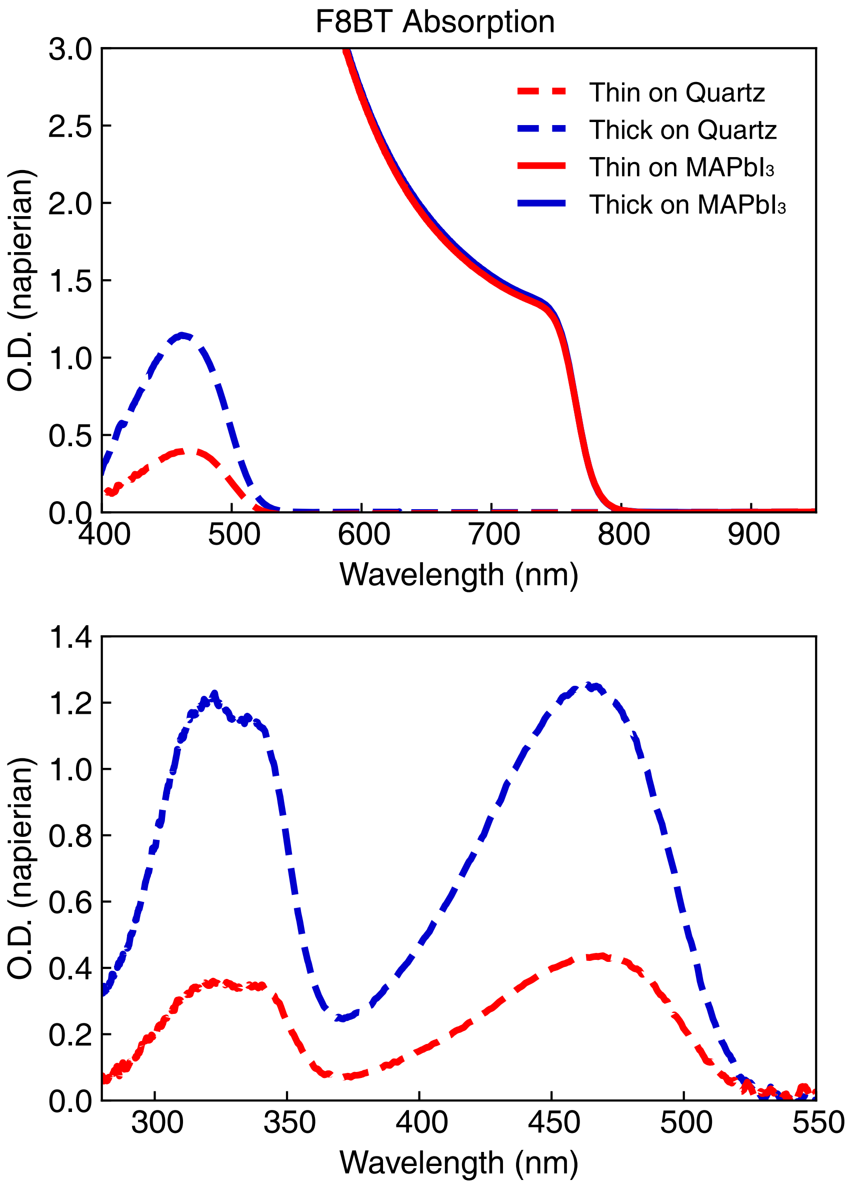

Supplementary Figure 2: F8BT absorption spectra. Top: Absorption of thin (red) and thick (blue) F8BT films on quartz (solid line) and on $MAPbI_3$:quartz (dashed line) substrates, measured using a NIR source and a Si detector. Bottom: Absorption of thin and thick F8BT films on quartz substrates, measured with a Deuterium lamp and a GaP detector.

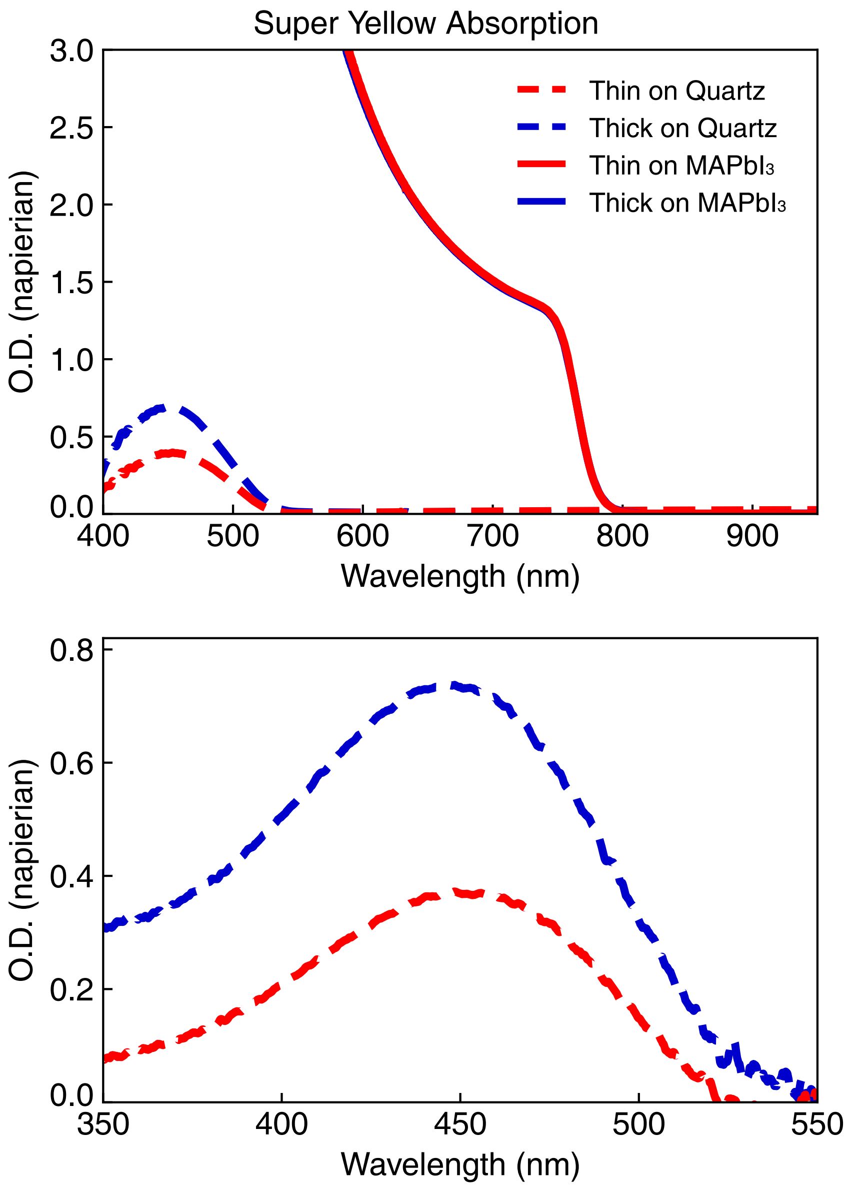

Supplementary Figure 3: Super Yellow absorption spectra. Top: Absorption of thin (red) and thick (blue) Super Yellow films on quartz (solid line) and on $MAPbI_3$:quartz (dashed line) substrates, measured with a NIR source and a Si detector. Bottom: Absorption of thin and thick Super Yellow films on quartz substrates, measured with a Deuterium lamp and a GaP detector.

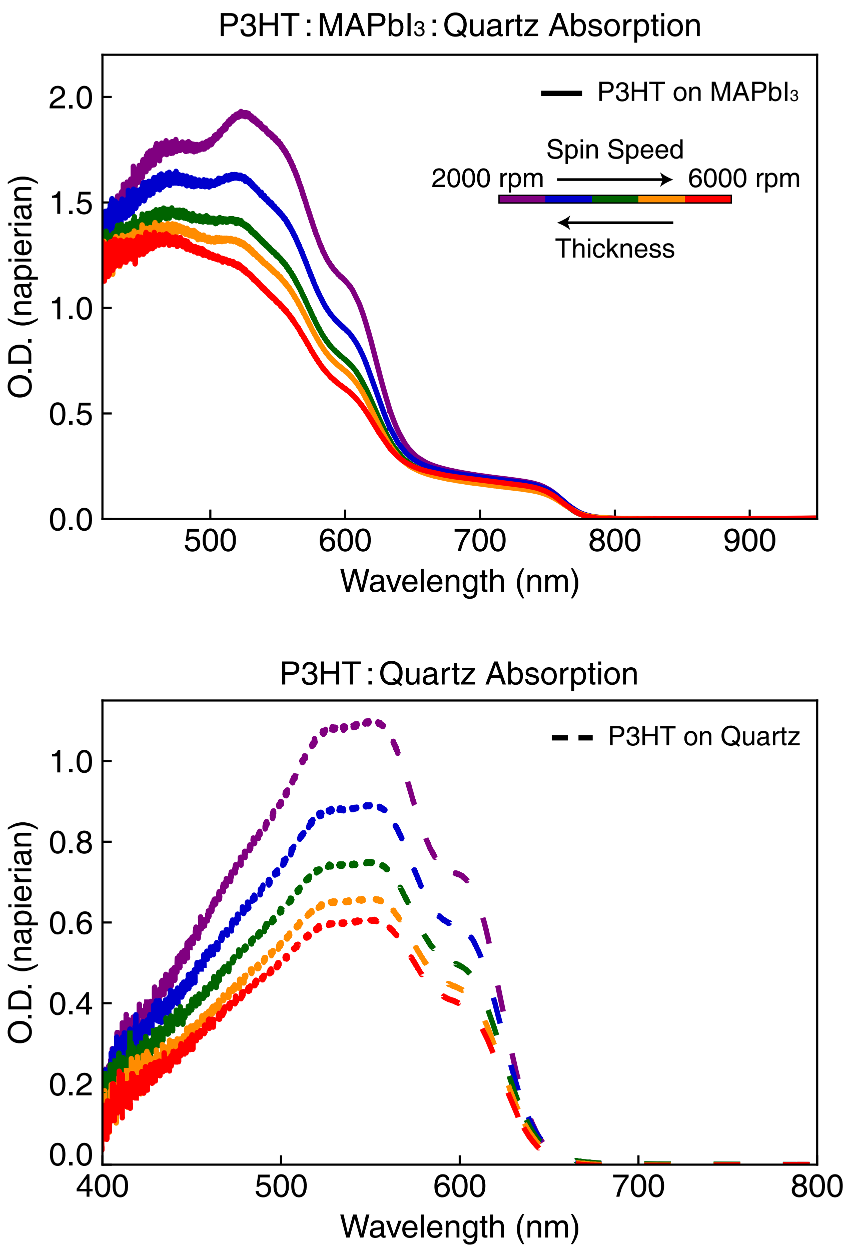

Supplementary Figure 4: P3HT absorption spectra. Top: Absorption spectra of P3HT films deposited with varying spin speeds, resulting in different film thicknesses (indicated through the color spectrum; spin speed changes in steps of 1000 rpm from 2000 rpm to 6000 rpm) on $MAPbI_3$:quartz (solid lines) substrates. Bottom: Absorption of P3HT films deposited with varying spin speed and resulting in different thicknesses on quartz (dashed lines) substrates, both measured with a NIR source and Si detector.

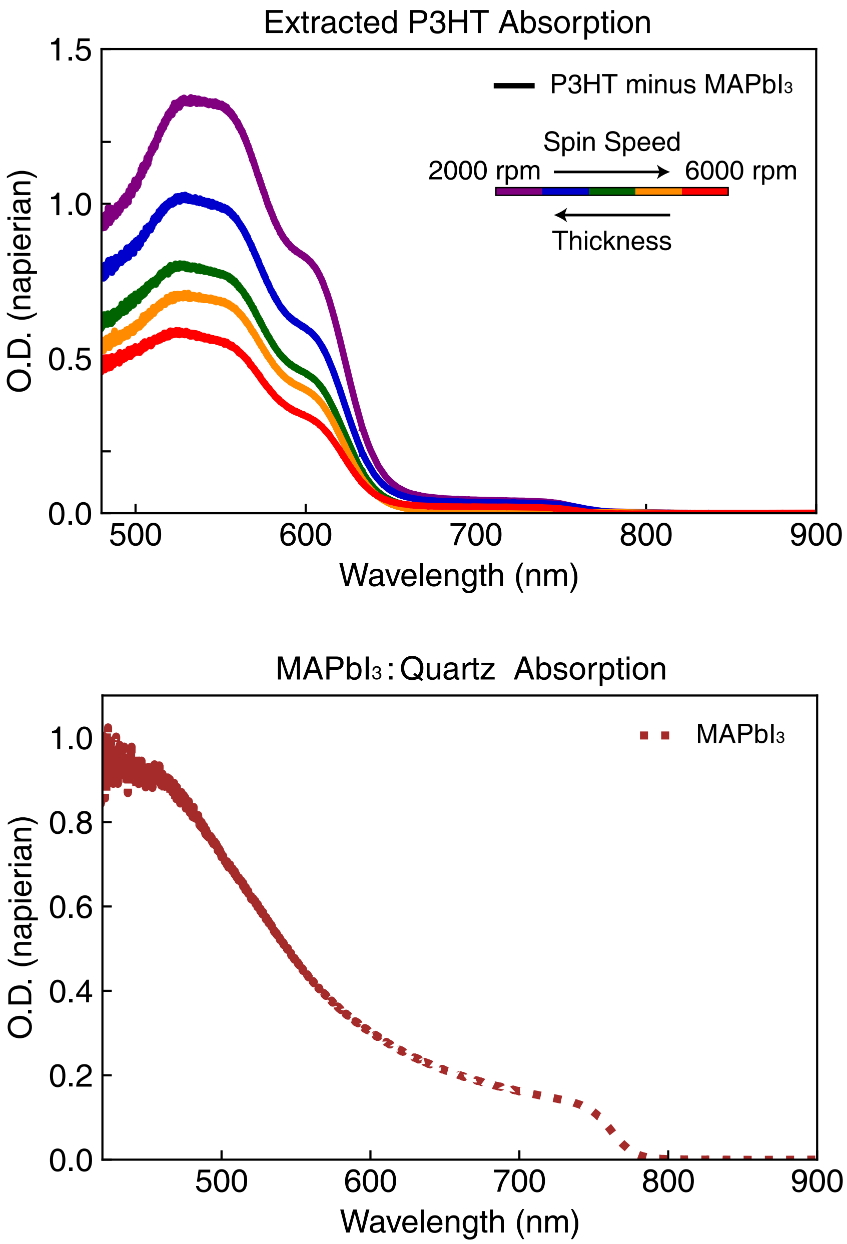

Supplementary Figure 5: P3HT absorption components for P3HT:MAPbI$_3$:quartz samples. Top: P3HT absorption component for P3HT:MAPbI$_3$:quartz samples (solid lines), with P3HT films deposited with varying spin speed resulting in different film thicknesses (indicated through the color spectrum as depicted). The absorption spectrum from the MAPbI$_3$ layer was subtracted from the overall P3HT:MAPbI$_3$:quartz spectra in order to obtain the P3HT-only components. Bottom: Absorption spectrum of a MAPbI$_3$:quartz sample (brown dotted line). Spectra were measured with a NIR source and Si detector.

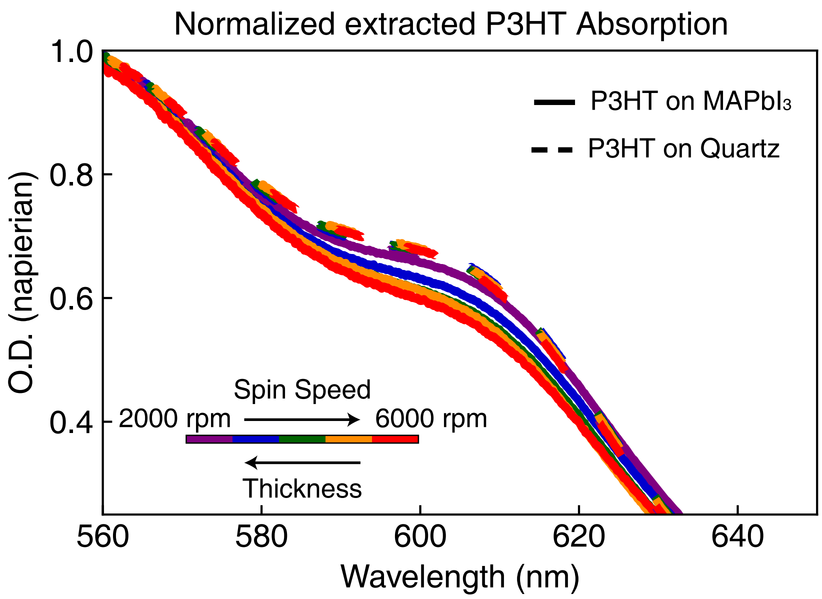

Supplementary Figure 6: Normalized and zoomed-in P3HT absorption spectra. Normalized (at 560 nm) P3HT component of absorption spectra determined for P3HT films deposited with varying spin speed and resulting in different thicknesses (indicated through the color spectrum as depicted) on quartz (dashed lines) and $MAPbI_3$:quartz (solid lines) substrates.

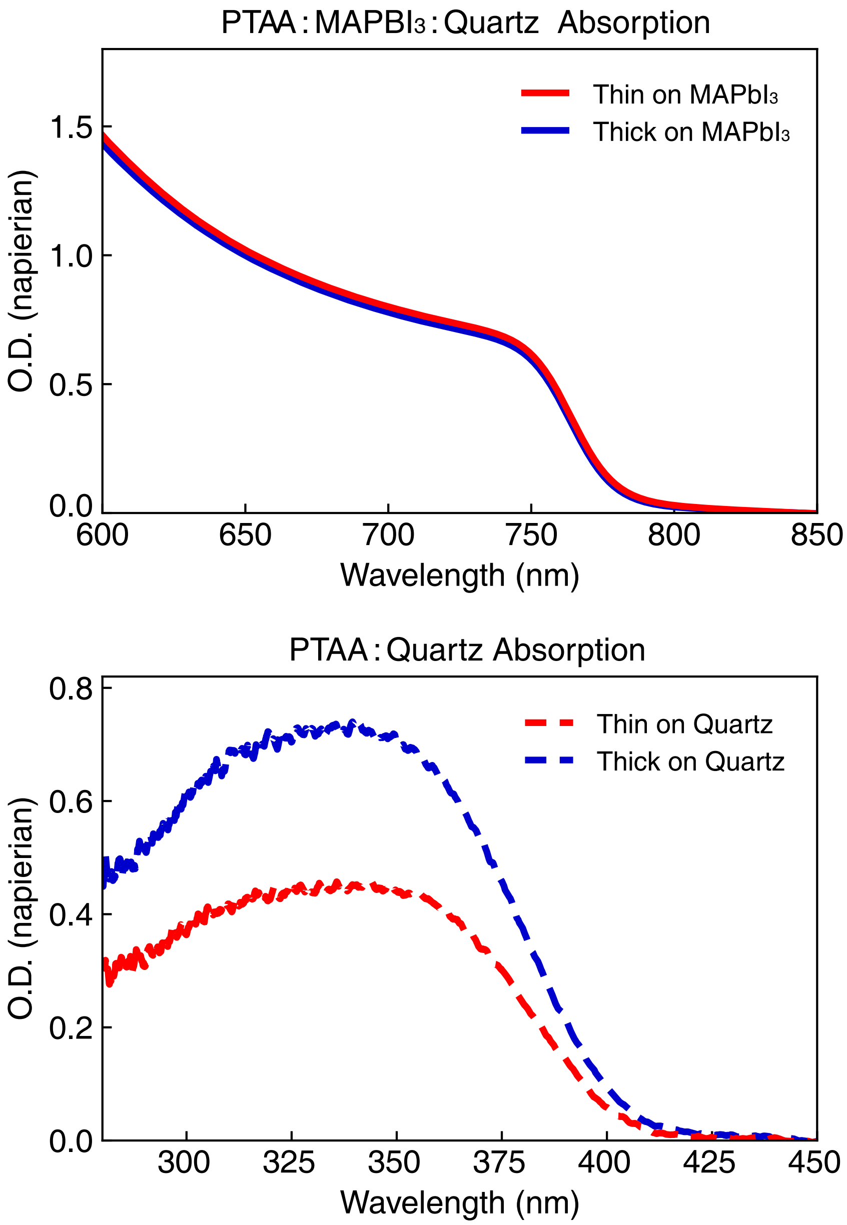

Supplementary Figure 7: PTAA absorption spectra. Top: Absorption of thin (red) and thick (blue) PTAA films on $MAPbI_3$:quartz (solid lines) substrates, measured with a NIR source and Si detector. Bottom: Absorption of thin and thick PTAA films on quartz (dashed lines), measured with a Deuterium lamp and GaP detector.

$MAPbI_3$ Absorption and Emission Spectra

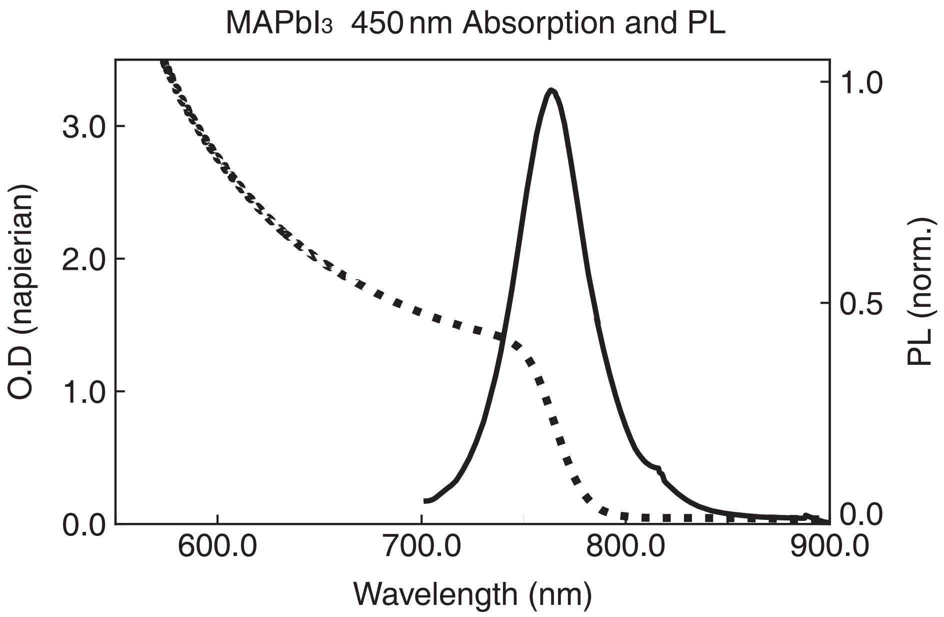

Supplementary Figure 8: 450 nm-thick $MAPbI_3$ layer. Absorption (dotted line) and PL spectra (solid line) of a 450 nm-thick $MAPbI_3$ layer on quartz, used as a substrate for F8BT and Super Yellow films. The absorption spectrum was measured with a NIR source and a Si detector. For the PL spectra, the film was excited at 400 nm with a fluence of the order of $20\,nJ\,cm^{-2}$.

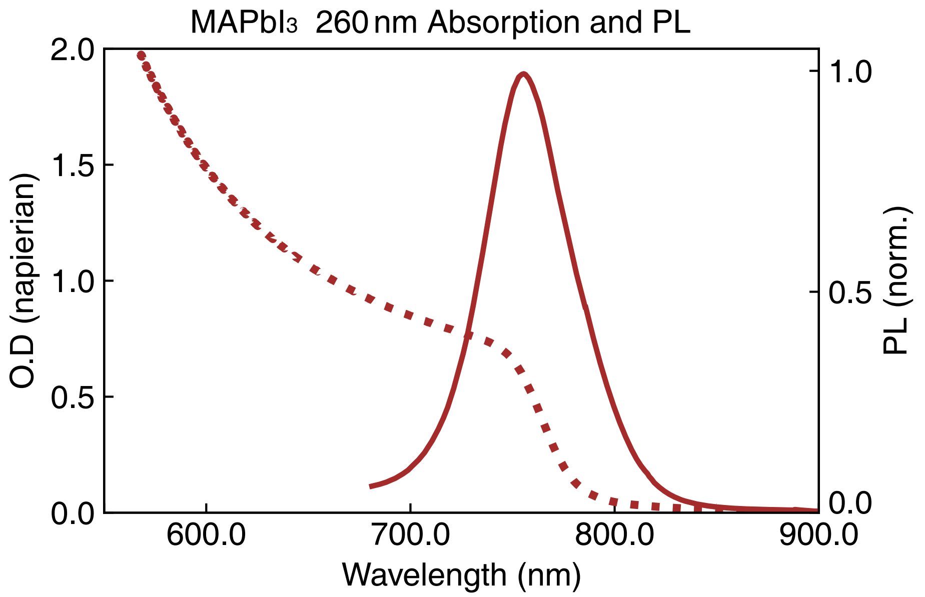

Supplementary Figure 9: 260 nm-thick $MAPbI_3$ layer. Absorption (dotted line) and PL spectra (solid line) of a 260 nm-thick $MAPbI_3$ layer on quartz, used as a substrate for PTAA films. The absorption was measured with a NIR source and a Si detector. For the PL spectra, the film was excited at 400 nm with a fluence of the order of $20\,nJ\,cm^{-2}$.

Photoluminescence Spectra of Polymer Films

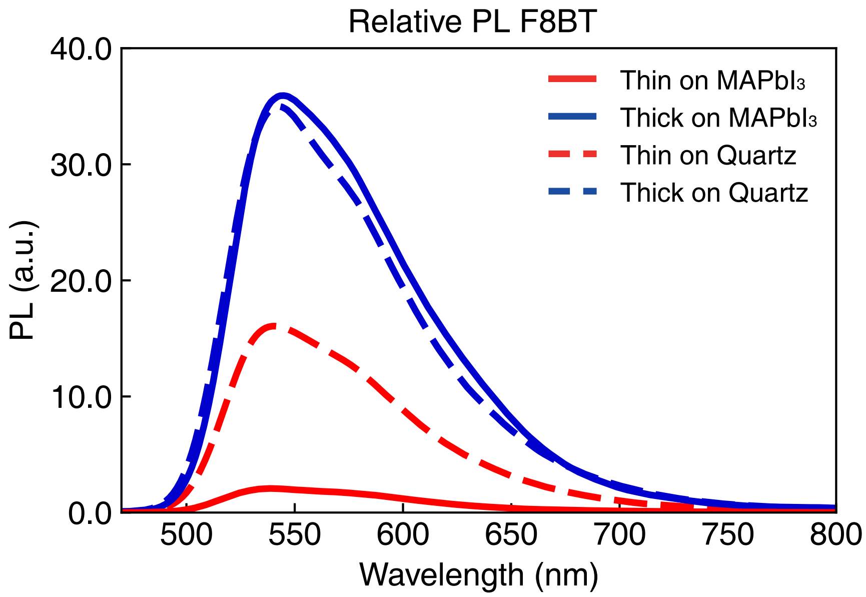

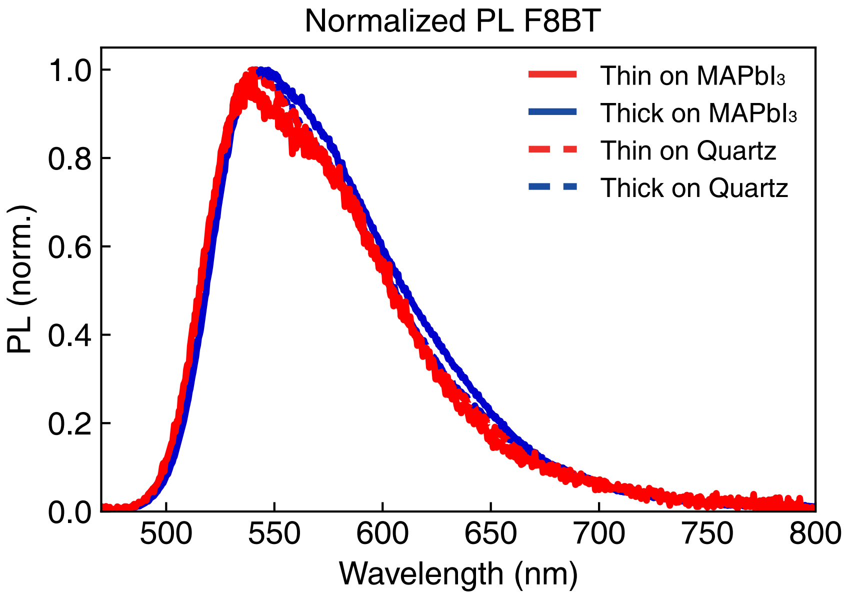

Supplementary Figure 10: F8BT emission spectra. PL spectra of F8BT for thick (blue) and thin (red) films on quartz (dashed line) and on $MAPbI_3$ (solid line). Top: Relative intensity of the PL. Bottom: Normalized PL Spectra. F8BT films were excited at 440 nm with a fluence of $2\,nJ\,cm^{-2}$. For the thin sample on $MAPbI_3$:quartz, reduced PL intensity is observed, which is a sign of energy transfer. No significant changes in PL lineshape were observed for films of different thickness of F8BT.

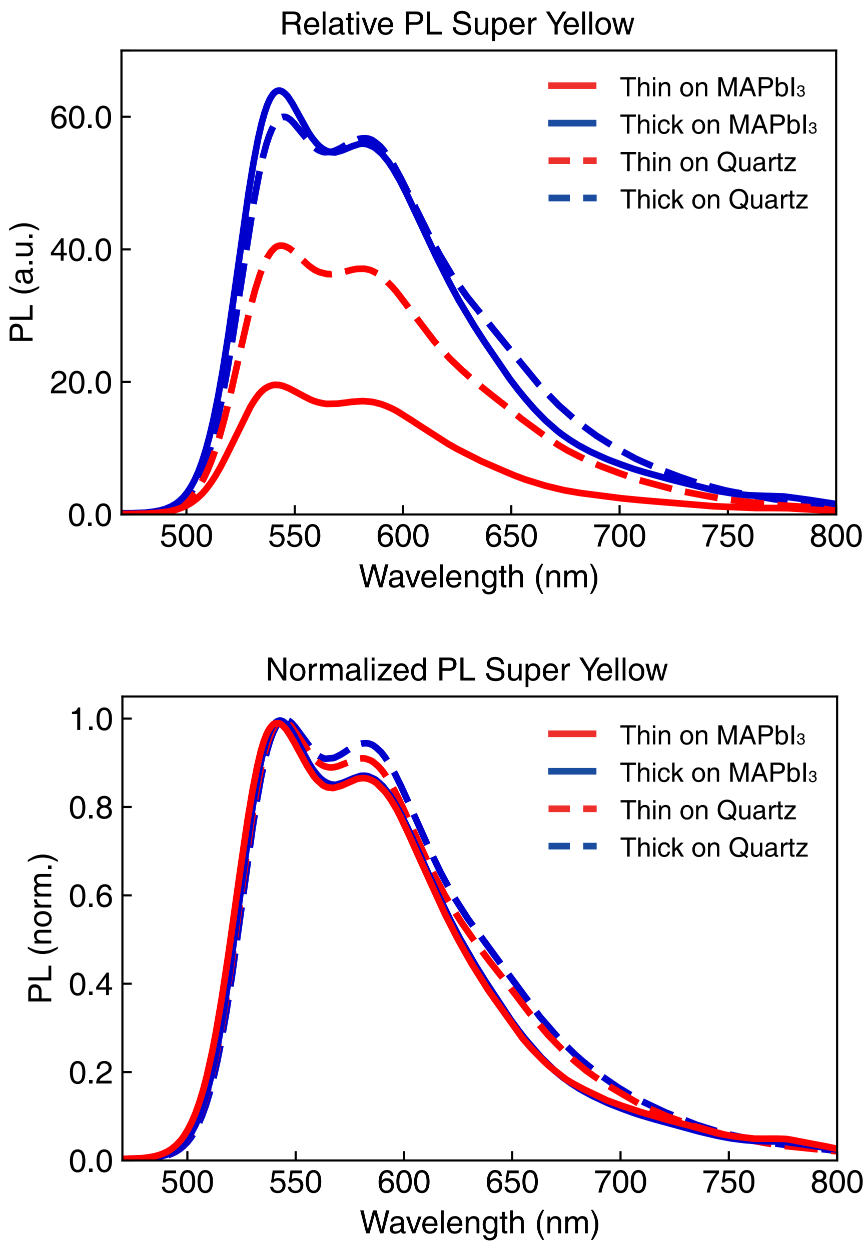

Supplementary Figure 11: Super Yellow emission spectra. PL spectra of Super Yellow for thick (blue) and thin (red) films on quartz (dashed line) and on $MAPbI_3$:quartz (solid line) substrates. Top: Relative intensity of the PL. Bottom: Normalized PL Spectra. Super Yellow films were excited at 440 nm with a fluence of $2\,nJ\,cm^{-2}$. For the thin sample on $MAPbI_3$:quartz, reduced PL intensity is observed, which is a sign of energy transfer. Slight changes in PL lineshape for the films of different thickness of Super Yellow are also observed, which might stem from to small morphological changes of the material when deposited on $MAPbI_3$.

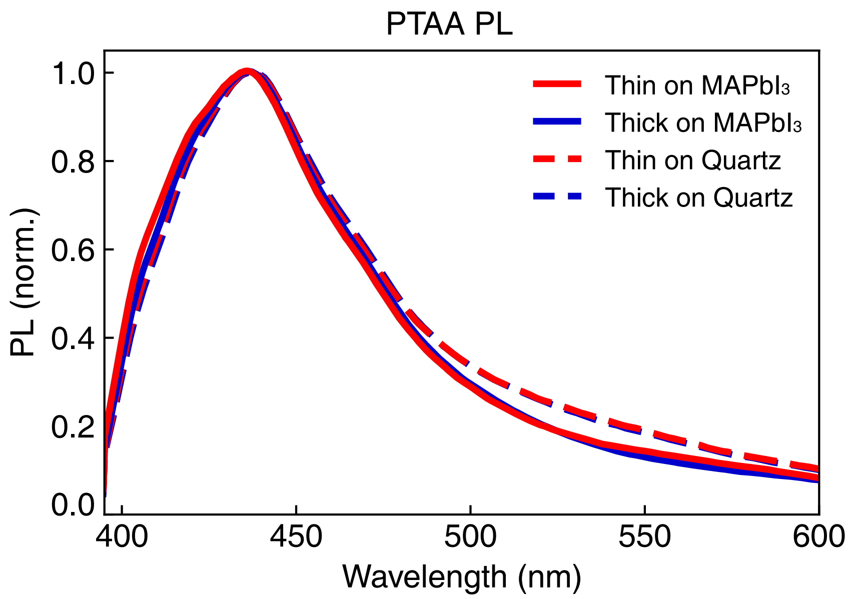

Supplementary Figure 12: PTAA emission spectra. Normalized PL spectra of PTAA for thick (blue) and thin (red) films on quartz (dashed line) and on $MAPbI_3$:quartz (solid line) substrates. PTAA was excited for PL, TCSPC and PLQE measurements at 360 nm with a fluence of the order of $1\,nJ\,cm^{-2}$. Here, only the normalized PL is shown as the PL intensity correlates with film thickness and no reduction in PL intensity is observed. Only very small, but insignificant changes are found for the PL lineshape with film thickness.

PL Decay Transients of Polymer Films

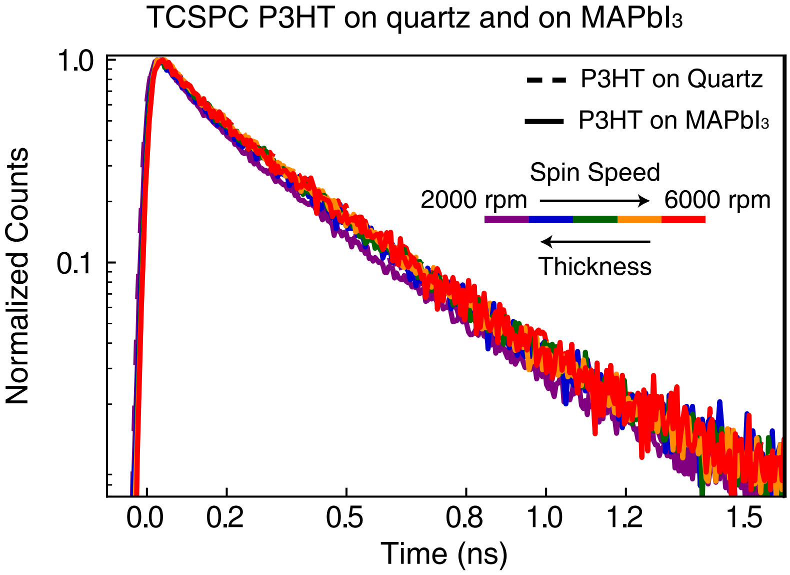

Supplementary Figure 13: Normalized PL transients of P3HT detected at 725 nm. Normalized TCSPC traces recorded for P3HT films deposited with different spin speeds resulting in different film thickness on quartz (dashed lines) and $MAPbI_3$:quartz (solid lines) substrates, detected at 725 nm. The thickness/spin speed is indicated through the color scheme as depicted. No evidence of energy transfer or any other influence of thickness or surface (quartz or $MAPbI_3$) was found for these films.

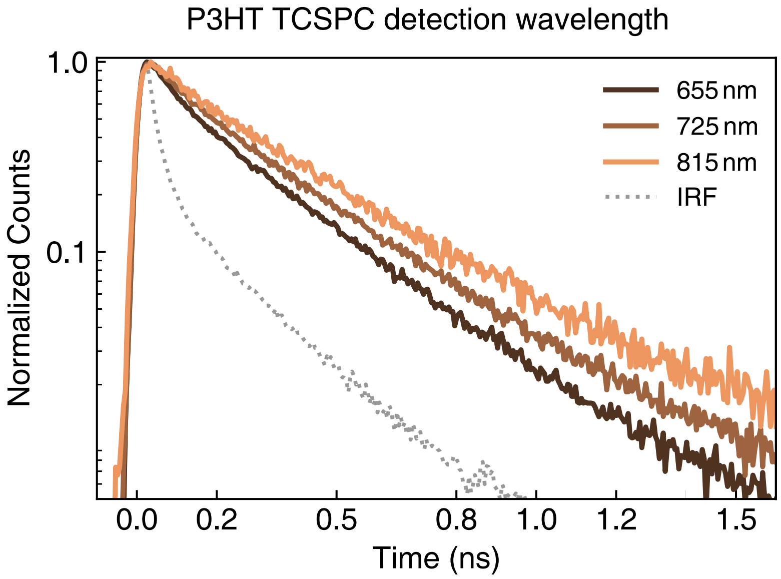

Supplementary Figure 14: Normalized PL transients of P3HT detected at various wavelengths. Normalized TCSPC traces recorded for P3HT films on quartz (spin speed: 4000 rpm, thickness: 52 nm), detected at three different wavelengths, which are indicated through their color as defined in the legend. Additionally, the instrumental response function is plotted as a grey dotted line. A dependence of the PL decay (see Fig. S14) on detection wavelengths is observed, which agrees with having different emissive species (disordered and aggregated) in the P3HT films.

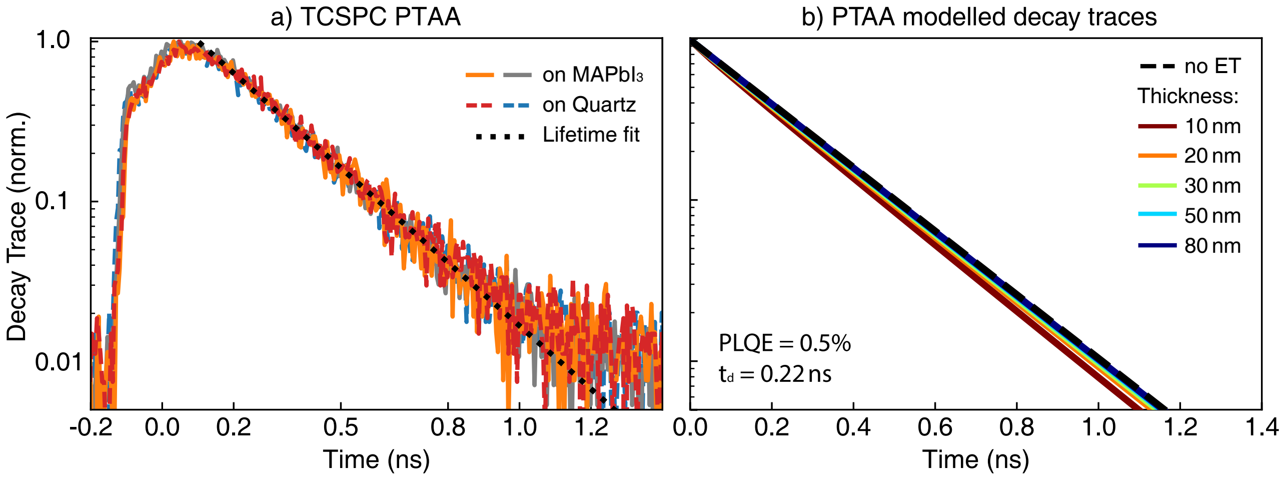

Supplementary Figure 15: PTAA PL transients. a) Normalized TCSPC traces of PTAA films of different thicknesses on quartz (dashed lines) and $MAPbI_3$:quartz substrates (solid lines), detected at 460 nm. No influence of either thickness or substrate on is seen. The mono-exponential fit to the decay transient is plotted on top of the data as black dotted curve. b) Modelled decay traces for PTAA films on $MAPbI_3$, taking into account energy transfer (ET) from the polymer to the $MAPbI_3$ layer, for polymer layer thicknesses between 10 nm and 80 nm (thickness indicated by color as depicted in the legend). The decay trace for a PTTA sample with no ET is shown as black dashed line. Values of the PLQE and decay lifetime as used in the model are indicated in the bottom left corner.

Absorption and Emission Spectra of Fluorescence Standard Rhodamine 6G

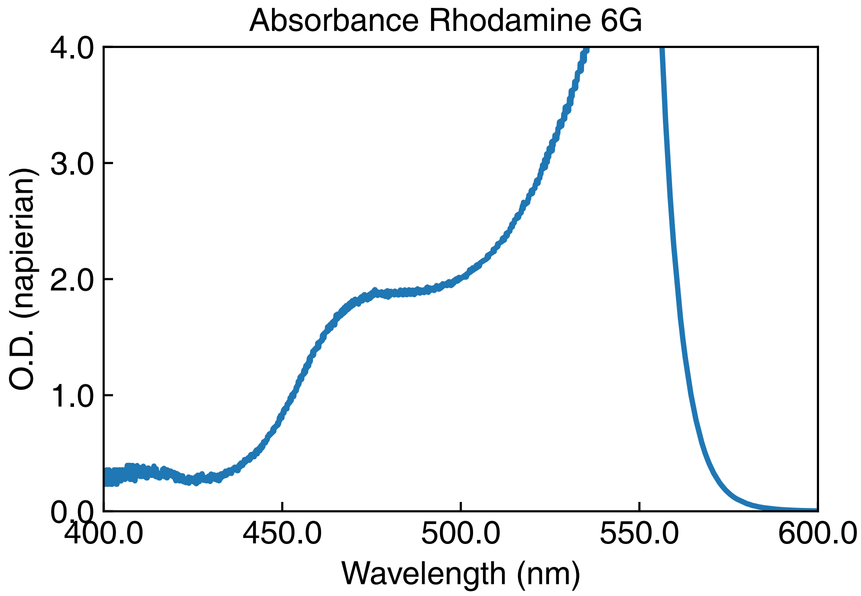

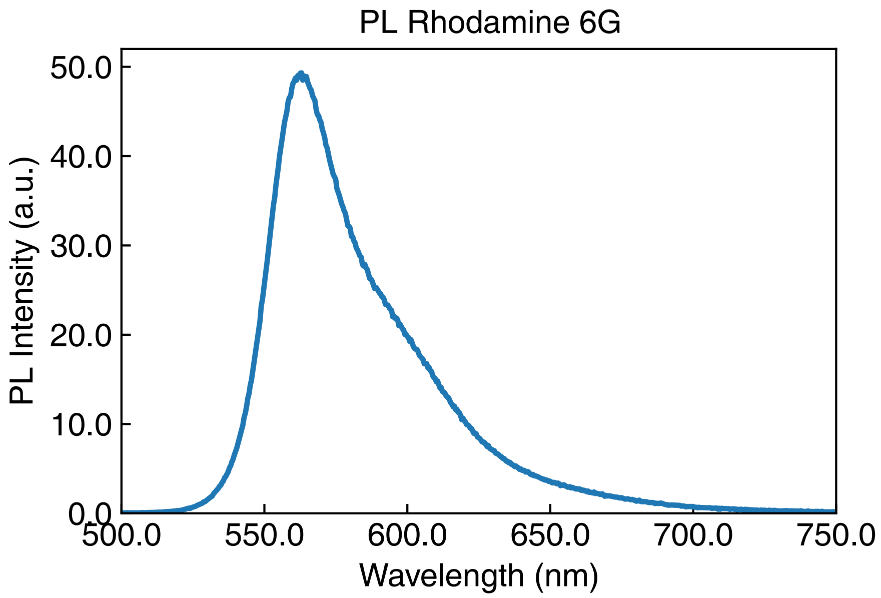

Supplementary Figure 16: Fluorescence Standard Rhodamine 6G. Absorbance and PL from a 0.8 mM Rhodamine 6G solution in ethanol are shown, which were used as a reference for PLQE determination of polymer films. Top: Absorbance spectrum of the solution, measured with an FTIR, using a NIR source and a Si detector. Bottom: PL spectrum of the Rhodamine 6G solution.

ET Efficiency

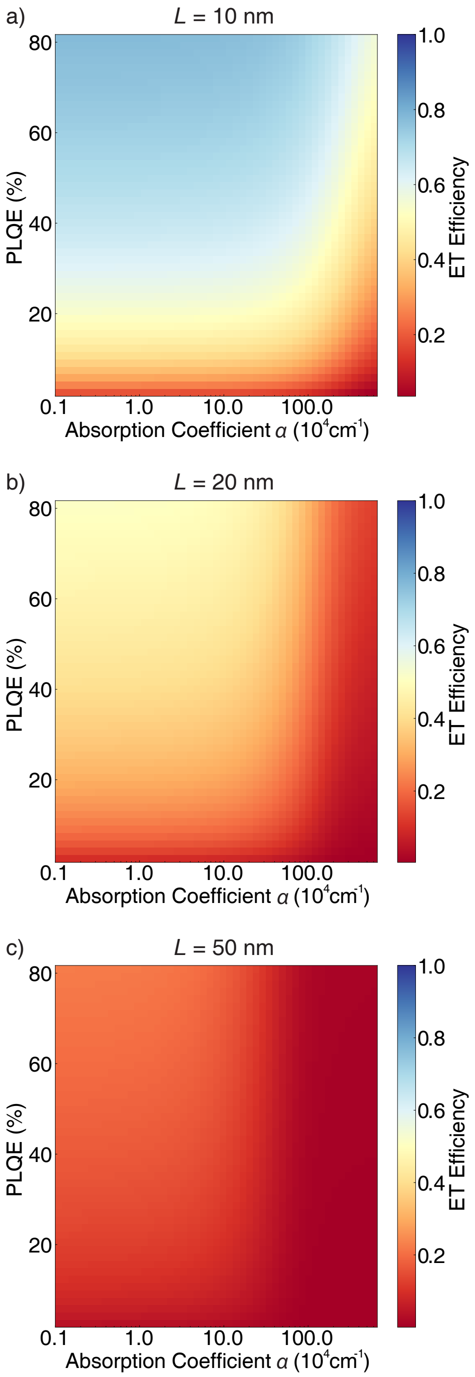

Supplementary Figure 17: False-color plots of ET efficiency modelled as function of α and PLQE. Efficiency of the ET between a photoexcited polymer layer and a $MAPbI_3$ layer underneath was modelled as described in the main article and is displayed as a function of the absorption coefficient α at the excitation wavelength, and the PLQE of polymer films, shown here for polymer films of a) 10 nm b) 20 nm c) 50 nm thickness.

Supplementary Tables

Material	E_g (eV)	IE (eV)	EA (eV)	HOMO (eV)	LUMO (eV)
$FASnCl_3$ [4]	3.55	7.33	3.83		
$MASnCl_3$ [4]	3.5	6.85	3.36		
$MAPbCl_3$ [4]	3.04	6.92	3.77		
$FAPbCl_3$ [4]	3.02	6.94	3.98		
$CsPbCl_3$ [4]	2.99	6.8	3.77		
$CsSnCl_3$ [4]	2.88	6.44	3.47		
$FASnBr_3$ [4]	2.63	6.23	3.6		
$CsPbBr_3$ [4]	2.31	6.53	4.17		
$MAPbBr_3$ [4]	2.30	6.6	4.25		
$FAPbBr_3$ [4]	2.25	6.7	4.51		
$MASnBr_3$ [4]	2.13	5.67	3.42		
$CsSnBr_3$ [4]	1.18	5.82	4.07		
$CsPbI_3$ [4]	1.72	6.25	4.47		
$MAPbI_3$ [4]	1.59	5.93	4.36		
$FAPbI_3$ [4]	1.51	6.24	4.74		
$CsSnI_3$ [4]	1.25	5.69	4.38		
$MASnI_3$ [4]	1.24	5.39	4.07		
$FASnI_3$ [4]	1.24	5.34	4.12		
Super Yellow [5]	2.5			5.2	2.7
F8BT [6]	2.3			5.8	3.5
P3HT [7]	2			5	3
PTAA [8]	2			5	3

Supplementary Table 1: Energy levels of a range of MHPs and the polymers investigated in this study. Excellent band alignment is needed for well-functioning MHP-based solar cells. This table presents ionization energy (IE), electron affinity (EA) and band gap energy E_g for a range of MHP materials as well as highest occupied molecular orbital (HOMO) and lowest unoccupied molecular orbital (LUMO) for the polymers investigated in this study, taken from literature references as indicated.

	F8BT	Super Yellow	P3HT	PTAA
thin polymer : quartz	30 nm	30 nm	42 nm	23 nm
thick polymer : quartz	80 nm	60 nm	77 nm	55 nm
thin polymer : $MAPbI_3$: quartz	25 nm	23 nm	40 nm	35 nm
thick polymer : $MAPbI_3$: quartz	78 nm	50 nm	92 nm	100 nm

Supplementary Table 2: Thickness of the polymer layers labelled as "thick" and "thin". This table lists film thickness of the polymer samples investigated throughout this study with respect to their labelling as "thick" and "thin".

	2000 rpm	3000 rpm	4000 rpm	5000 rpm	6000 rpm
polymer : quartz	77 nm	62 nm	52 nm	46 nm	42 nm
polymer : $MAPbI_3$: quartz	92 nm	70 nm	55 nm	48 nm	40 nm

Supplementary Table 3: Thickness of the P3HT layers deposited from spin casting with varying spin speed. This table lists film thickness of the P3HT layers as achieved through changes in spin speed (2000–6000 rpm, in 1000 rpm steps) during their fabrication from spin-casting.

	F8BT	Super Yellow	P3HT	PTAA
Lifetime	0.85 ns	1 ns	0.3 ns	0.22 ns
Excitation Wavelength	400 nm	400 nm	460 nm	360 nm
Detection Wavelength	540 nm	600 nm	725 nm	460 nm
Absorption Coefficient	$0.013\,nm^{-1}$	$0.013\,nm^{-1}$	$0.008\,nm^{-1}$	$0.015\,nm^{-1}$
PLQE	$71 \pm 1\%$	$26 \pm 3\%$	$1 \pm 0.3\%$	$0.5 \pm 0.03\%$

Supplementary Table 4: PL lifetime, excitation and detection wavelength, absorption coefficient at the excitation wavelength and PLQE for the different polymers. This table lists optoelectronic properties (lifetime, absorption coefficient and PLQE) and the experimental settings (excitation and detection wavelength) used to determine them for the four polymers investigated in this study.

Supplementary Notes

Supplementary Note 1: Fluorescence Decay and ET Efficiency Model

In the main text, we describe our observation of accelerated PL decay for high PLQE polymers (Super Yellow and F8BT) on $MAPbI_3$:quartz compared to their PL decay on quartz. No change in PL decay time was detected for low PLQE polymers on $MAPbI_3$:quartz. The faster PL decay observed mainly for the thin (20–30 nm) layers of luminescent polymers suggests that energy transfer (ET) may occur between the polymer and the $MAPbI_3$ as described by Förster resonant energy transfer (FRET). The occurrence of ET is further supported by the observation of lower PL intensity in these samples (see above). In this section, we apply FRET to model ET between the polymer and the $MAPbI_3$. In a first step, the application of FRET to a two-layer geometry is outlined. The theory leads to an expression for the PL decay as a function of time, which is fitted to the PL decay traces in a second step. Using the results from the first two steps, the efficiency of ET from polymer to $MAPbI_3$ is calculated based on parameters of the polymer layer in a third step.

1.1 FRET in a Two-Layer Geometry

A theory of resonant ET from an energy donor to an energy acceptor based on their dipole-dipole interactions was developed by Theodor Förster [9]. The rate of the ET, $k(r)$ as a function of donor-acceptor distance r, donor lifetime τ_d, and the Förster radius R_0 is expressed as:

$$k(r) = \frac{1}{\tau_\mathrm{d}}\Big(\frac{R_0}{r}\Big)^6 \tag{S.2}$$

with

$$R_0 = \Big(\frac{9\ \ln(10)\ \kappa^2\ \phi_\mathrm{d}}{128\ \pi^5\ N_\mathrm{A}\ n^4}J\Big)^{1/6}, \tag{S.3}$$

where κ^2 accounts for the geometric orientation of the donor and acceptor dipoles, ϕ_d is the PLQE of a purely donor sample, N_A denotes the Avogadro constant, n is the refractive index and J is the overlap integral between donor emission and acceptor absorption [9, 10]. R_0 has units of nm if J is entered in units of $\frac{\mathrm{dm^3 nm^4}}{\mathrm{mol\,cm}}$ [11]. R_0 defines the distance at which

any other pathway of de-excitation of the donor is as likely as de-excitation via FRET. The orientation factor will be assumed to be $\kappa = 0.845\sqrt{2/3}$ for further discussions, which assumes random but fixed relative orientations of donor and acceptor dipoles within a solid film [12]. Förster's original expression for the rate of ET between donor and acceptor as stated above was derived for ET within a solution (3-dimensional space) with uniform donor and acceptor concentrations [13, 14]. As FRET has become an important tool for measuring nano-scale distances, the theory has been extended to different applications and sample geometries [15, 16, 17, 18, 19, 20, 21].

A more general expression, which can also take into account the effect of restricted geometries, was formulated by Klafter and Blumen [19] ("KB-equation"), who introduced a dimensionality parameter β, which is linked to spatial dimensions as $\beta = d/6$ (in case that no further spatial restrictions but dimensionality exist) to describe the donor decay $I_{\mathrm{d}}(t)$ as:

$$I_{\mathrm{d}}(t) \;=\; e^{-t/\tau_{\mathrm{d}} \;-\; p\;(t/\tau_{\mathrm{d}})^{\beta}} \tag{S.4}$$

where p is a parameter linked to the local acceptor concentration and also dependent on β. This formalism has been further extended to also incorporate arbitrary distributions of donors and acceptors of which a derivation and overview is given in Refs. 22, 21. As main result, the donor decay $I_{\mathrm{d}}(t)$ as a function of time for ET between donors and acceptors, integrated over their positions $\mathbf{r_d}$ and $\mathbf{r_a}$ within a restricted volume, is given by:

$$I_{\mathrm{d}}(t) \;=\; e^{-t/\tau_{\mathrm{d}}} \int_V C_{\mathrm{d}}(\mathbf{r_d})\; \varphi(t,\mathbf{r_d})\; d\mathbf{r_d} \tag{S.5}$$

with:

$$\varphi(t,\mathbf{r_d}) \;=\; \exp\Big(-\int_V C_{\mathrm{a}}(\mathbf{r_a})\;[1 - e^{-t\;k(|\mathbf{r_a}-\mathbf{r_d})|}]\;d\mathbf{r_a}\Big), \tag{S.6}$$

where $C_{\mathrm{d}}(\mathbf{r_d})$ and $C_{\mathrm{a}}(\mathbf{r_a})$ denote the heterogeneous donor and acceptor concentrations, respectively. Yekta et al. [23] applied the formalism to systems with planar geometry and used it to derive analytic expressions for fluorescence decay inter alia for the situation of "parallel slabs of donor and acceptor touching at a sharp interface". Their approach will

be outlined in the following and in the final step we will introduce a non-uniform donor concentration $C_\mathrm{d}(z)$ to account for the fact that the number of initially excited donors as a function of film depth z is proportional to a Beer-Lambert profile.

For systems with planar symmetry, the donor and acceptor concentrations, $C_\mathrm{d}(z)$ and $C_\mathrm{a}(z)$ respectively, vary along a single spatial direction, here denoted as z-axis. For any thin vertical slice of the film, the donor and acceptor concentrations are assumed to be constant and Eqns. S.5 and S.6 can be rewritten as:

$$I_\mathrm{d}(t) \;=\; e^{-t/\tau_\mathrm{d}} \int C_\mathrm{d}(z)\;\varphi(z,t)\;dz \tag{S.7}$$

$$\varphi(z,t) \;=\; \exp\Big(-\int_V C_\mathrm{a}(z)\;[1-e^{-t\;k(r)}]\;dV\Big) \tag{S.8}$$

Geometrical considerations lead to the general expression for $I_\mathrm{d}(t)$ for systems with planar symmetry:

$$I_\mathrm{d}(t) \;=\; e^{-t/\tau_\mathrm{d}} \int_{-\infty}^{+\infty} C_\mathrm{d}(z)\;\varphi(z,t)\;dz \tag{S.9}$$

$$\varphi(z,t) \;=\; \exp\Big(-h(z,t)\Big) \tag{S.10}$$

$$h(z,t) \;=\; 2\pi \int_0^\infty r\,\Big(1-e^{-t/\tau_\mathrm{d}\;(R_0/r)^6}\Big)\int_{\mathrm{z-r}}^{z+r} C_\mathrm{a}(\hat{z})\;d\hat{z}\;dr. \tag{S.11}$$

The planar system of interest for us consists of two extended layers touching at an interface. In our case, one layer consists of a luminescent polymer donating energy to the perovskite acceptors in the second layer. In order to arrive at a numerical expression for this situation, the interface is positioned at $z=0$, the donor layer extends from $z=0$ to L and the extended acceptor layer spans from $z=0$ to $-\infty$. The assumption of an infinite acceptor layer is well justified as most of the ET happens within a few R_0, which is much smaller than the thickness of the $\mathrm{MAPbI_3}$ layers we are considering. The concentration of donors and acceptors within each respective extended layer can be assumed to be uniform: C_d and C_a. However, the number of initially excited donors will be assumed to follow a Beer-Lambert profile and is therefore not uniform. From these assumptions and geometrical definitions, the

concentrations can be written as:

$$C_\mathrm{d}(z) \;=\; C_\mathrm{d}\; e^{-\alpha\;(L-z)} \tag{S.12}$$

$$C_\mathrm{a}(z) \;=\; C_\mathrm{a}\; H(-z) \tag{S.13}$$

where α is the absorption coefficient of the polymer at the excitation wavelength used for the donor decay measurements and $H(x)$ denotes the Heaviside function (0 for $x < 0$ and 1 for $x \geq 0$). Using these definitions, the donor decay can be written as:

$$I_\mathrm{d}(t) \;=\; e^{-t/\tau_\mathrm{d}}\; C_\mathrm{d} \int_0^L e^{-\alpha\;(L-z)}\; \varphi(z,t)\; dz \tag{S.14}$$

$$\varphi(z,t) \;=\; \exp\Big(-\frac{\pi}{3}\; C_\mathrm{a}\; R_0^3\; \Big(\frac{t}{\tau_\mathrm{d}}\Big)^{\frac{1}{2}}\; [T^{-\frac{1}{2}}(1-e^{-T}) + 2\gamma(\tfrac{1}{2},T) - 3T^{-\frac{1}{6}}\; \gamma(\tfrac{2}{3},T)]\Big) \tag{S.15}$$

where the substitution

$$T \;=\; \frac{t}{\tau_\mathrm{d}}\; \Big(\frac{R_0}{z}\Big)^6 \tag{S.16}$$

has been made and $\gamma(n,T)$ is the incomplete gamma function, defined as:

$$\gamma(n,T) \;=\; \int_0^T u^{n-1}\; e^{-u}\; du. \tag{S.17}$$

1.2 PL Decay Modelling

Equations 1 to 4 in the main text enable modelling of the PL decay data from the polymers on $MAPbI_3$:quartz. The modelling was performed in two steps: First, we used the expression for $I_\mathrm{d}(t)$ in Eq. S.14 and R_0 as a fitting parameter to fit the data from SuperYellow:$MAPbI_3$:quartz. In a second step, we assume that the difference in R_0 between the polymers is a function of PLQE only (discussion below) and use the measured PLQE values to obtain $I_\mathrm{d}(t)$ through Eq. S.14 for F8BT, P3HT and PTAA.

The parameters for calculating $I_\mathrm{d}(t)$ are: the donor lifetime τ_d, donor (polymer) and acceptor ($MAPbI_3$) concentration C_d and C_a, polymer film thickness L, Förster radius R_0 and absorption coefficient of the polymer α. We will discuss and analyze these parameters and how they were determined in the following.

- The **donor lifetime** τ_d in absence of any acceptors was obtained from polymer films on a quartz substrate. A mono-exponential decay was fitted to the TCSPC data over the following time ranges: 0.1–3 ns (F8BT and Super Yellow), 0.1–1.5 ns (P3HT) t_0 was set to the peak of the rise of the TCSPC data. The fits are indicated as dashed lines in Fig. 2 and the obtained lifetimes for the different polymers are listed in Table S4. In addition, we fitted a mono-exponential decay at early times (<1.5 ns) and at later times (1.5–4 ns) of the PL transient for F8BT and SuperYellow, in order to test our assumption of a mono-exponential decay. Deviations from the donor lifetimes obtained are small (7% for F8BT and 13% for SuperYellow) and do not influence the modelled ET efficiency. This ensures that our assumption of a mono-exponential PL decay does not noticeably influence the ET efficiency we obtain through our modelling.

- The **film thickness** L of the polymer film on $MAPbI_3$ was obtained from DEKTAK measurements and is used as a specific input parameter. The values for the polymer films used in this study are given in Table S2.

- The **donor concentration** C_d (in units of a number density) is assumed to be uniform within the polymer film and is used as a scaling factor only. C_d scales the overall PL intensity in Eq. S.14. The fitting is performed with normalized PL decay traces, which are not determined in absolute terms. Therefore, we do not assign a specific value to C_d. However, the concentration of *excited* donors as a function of film penetration depth, $C_\mathrm{d}(z)$ was assumed to follow Beer's law (see Eq. S.12), which was incorporated into the expression for $I_\mathrm{d}(t)$ in Eq. S.14.

- The **absorption coefficient** α was obtained from absorption data (see above) at the excitation wavelength for the PL decay measurements (see Table S4) and from the polymer film thickness (see Table S2). It is a fixed input parameter to the model, specific to each polymer. The values are listed in Table S4 in units of nm^{-1} as entered in $I_\mathrm{d}(t)$. The unit nm^{-1} is used for consistency as the film thickness L and the film penetration depth z are entered in nm in Eq. S.14.

- The **acceptor ($\mathbf{MAPbI_3}$) concentration** C_a can be expressed a function of the refractive index n, PLQE ϕ_d, the spectral overlap J and the Förster Radius R_0. These physical quantities therefore become input parameters for modelling the PL decay data and will be additionally discussed. First, however, an equation to express C_a in terms of these quantities will be derived. C_a has units of nm^{-3} as we enter R_0 in units of nm in Eq. S.15. The Förster radius is defined in Eq. S.3 and can be rewritten in terms of units as [10, 11, 24]:

$$\frac{R_0}{\mathrm{nm}} = 0.02108 \left(\frac{\kappa^2\ \phi_\mathrm{d}}{n^4}\left[\frac{J}{\mathrm{mol}^{-1}\mathrm{dm}^3\mathrm{cm}^{-1}\mathrm{nm}^4}\right]\right)^{1/6}, \quad \text{(S.18)}$$

with the overlap integral J as a function of wavelength λ in nm. J is given as:

$$J = \int I_\mathrm{d}(\lambda)\epsilon_\mathrm{a}(\lambda)\lambda^4\ d\lambda, \quad \text{(S.19)}$$

where $I_\mathrm{d}(\lambda)$ is the normalized donor emission and $\epsilon_\mathrm{a}(\lambda)$ is the acceptor extinction coefficient.

$$\epsilon_\mathrm{a}(\lambda) = \frac{\log_{10}(e)}{C_\mathrm{a,molar}}\ \alpha_\mathrm{a}(\lambda), \quad \text{(S.20)}$$

where $\alpha_\mathrm{a}(\lambda)$ is the absorption coefficient as a function of wavelength in units of cm^{-1} and

$$C_\mathrm{a,molar} = \frac{\hat{C}_\mathrm{a}}{N_\mathrm{A}} \quad \text{(S.21)}$$

is the molar acceptor concentration in units of $\mathrm{mol/dm}^3$ and $\hat{C}_\mathrm{a}$ denotes the acceptor concentration in units of dm^{-3}. For C_a in in units of nm^{-3}, it follows:

$$C_\mathrm{a} = N_\mathrm{A}\ C_\mathrm{a,molar}\ 10^{-24} = 0.6022\ C_\mathrm{a,molar}. \quad \text{(S.22)}$$

In addition to the overlap integral J, the Förster radius R_0 in Eq S.18 is determined by the refractive index n, the orientation factor κ and PLQE ϕ_d. The orientation factor κ is fixed for all samples (see above) and we introduce the constant $k = \kappa^2/n^4$. Using

these definitions, we rewrite Eq. S.18 as:

$$R_0^6 \;=\; k\;\phi_{\rm d}\;J \;=\; k\;\phi_{\rm d}\,\frac{1}{C_{\rm a,molar}}\;\hat{J} \qquad \text{(S.23)}$$

with

$$\hat{J} \;=\; C_{\rm a,molar}\;J \;=\; \log_{10}(e)\int I_{\rm d}(\lambda)\alpha_{\rm a}(\lambda)\lambda^4\;d\lambda. \qquad \text{(S.24)}$$

Using the above, we can conclude with an expression for $C_{\rm a}$ as a function of R_0, $\phi_{\rm d}$, $\hat{J}$ and k:

$$C_{\rm a} \;=\; 0.6022\,\frac{k\;\phi_{\rm d}\;\hat{J}}{R_0^6}. \qquad \text{(S.25)}$$

Equation S.25 expresses the acceptor concentration $C_{\rm a}$ as a function of the refractive index n (through k), PLQE $\phi_{\rm d}$, the modified overlap $\hat{J}$ and the Förster Radius R_0. These physical quantities become additional input parameters for modelling the PL decay data and will be discussed in the following:

- The **refractive index** n refers in original FRET theory to the solution in which donors and acceptors were dissolved [10, 9], which no longer applies for the case of a two-solid-layers system. For films of π-conjugated polymers, an effective refractive index of $n_{\rm poly} = 1.6$ can be assumed [25, 26, 27] and $MAPbI_3$ has a refractive index of $n_{\rm MAPbI_3} = 2.5$ at the wavelengths at which the polymers emit [28]. We use the average value from the polymer and the perovskite of $n = 2.05$ in our calculations assuming that the refractive indexes of both media play an averaged role for the ET process across their interface.

- The modified **spectral overlap** $\hat{J}$ between donor emission and acceptor absorption is defined through Eq. S.24. $\hat{J}$ was calculated from $MAPbI_3$ absorption (Supplementary Figures S14 and S15) and polymer emission data (Supplementary Figures S7–S9). $\hat{J} = 3.4 \times 10^{14}\,{\rm nm}^3$ was calculated for Super Yellow and entered in the calculation of $C_{\rm a}$ (as in Eq. S.25) when fitting the PL decay data from SuperYellow:$MAPbI_3$:quartz.

- The **Förster radius** R_0 is defined in Equations S.3 and S.18. As mentioned above, modelling the PL decay of the polymers was done in two steps: In a first step, R_0 was used as a fitting parameter in Eqs. S.14 to S.17 to fit the PL decay data from SuperYellow:$MAPbI_3$:quartz to obtain R_0^{SY} and through Eq. S.25 also C_a (result from fitting: $R_0^{SY} = 8.5 \pm 0.6$ nm, $C_a = 0.0016\,\text{nm}^{-3}$). In a second step, $I_d(t)$ of the other polymers is modelled using Eqs. S.14 to S.17 with $C_a = 0.0016\,\text{nm}^{-3}$ as a global input parameter and

$$R_0^{\text{polymer}} = R_0^{\text{SY}} \left(\frac{\phi_{\text{polymer}}}{\phi_{\text{SY}}}\right)^{1/6} \tag{S.26}$$

as a polymer specific input parameter. In this step, we assume the polymer PLQE to be the most sensitive parameter in determining R_0 between the different polymers to describe the ET from the polymer to the $MAPbI_3$. R_0 (see Eq. S.18) is determined by the orientation factor κ, PLQE ϕ_d, the refractive index n and the spectral overlap J. κ is a fixed parameter and n is assumed to be the same for the investigated polymers. The other parameter next to ϕ_d which might change between the polymers, is the overlap integral J (see Eq. S.19) or, alternatively, $\hat{J}$, as defined in Eq. S.24. The spectral overlap is determined from normalized donor emission and acceptor absorption. All polymers were deposited on $MAPbI_3$, which acts as the acceptor. Therefore, the acceptor absorption represented through the extinction coefficient in J or the absorption coefficient in $\hat{J}$ in the overlap integral remains unchanged in any case. Additionally, there is (almost) full overlap between polymer emission and $MAPbI_3$ absorption for all polymers investigated. Differences in the overlap integral would therefore only stem from the wavelength-dependence of the normalized donor emission. The assumption of determining R_0^{polymer} through change in PLQE only is accordingly supported by the computed values of $\hat{J}$ for Super Yellow, F8BT and P3HT: $\frac{\hat{J}}{10^{14}\ \text{nm}^3} = 3.4$ (Super Yellow), 3.3 (F8BT) and 3.0 (P3HT, which has slightly less overlap). The PLQE values for these polymers range from 1% to 71%, which is clearly a much greater difference.

The error on the Förster radius and on the modelled PL decay data was determined from uncertainties in the measured input parameters ϕ_d (see Table S4) and L (10%). The error on R_0^{SY} was estimated from fitting the PL decay data with the upper and lower boundaries for ϕ_{SY} and L_{SY}. The error in the modelled PL decay data from the other polymers was then estimated from the resulting R_0^{SY}, polymer PLQE and polymer thickness. The error estimations are depicted as shaded area around the data in Fig. 1 in the main text.
To ensure that our approach of fitting the decay transients for SuperYellow:$MAPbI_3$:quartz to obtain the global model input parameters R_0^{SY} and C_a as a first step of the PL decay modelling is indeed sensible, we confirmed comparable outputs from a secondary approach. Here we alternatively fitted the PL transient data for SuperYellow:$MAPbI_3$:quartz and F8BT:$MAPbI_3$:quartz simultaneously to obtain these global parameters (using Eq. S.26 to relate R_0^{SY} and R_0^{F8BT}). The results for the ET efficiency calculated with this fitting approach show only very small deviation (+/- 1%) from the results obtained by fitting the data from SuperYellow:$MAPbI_3$:quartz only, which strongly supports our approach.

1.3 Calculation of ET Efficiency

The ET efficiency η_{ET} is defined in the main text in Eq. 6, here we briefly describe the numerical calculation of η_{ET}. F_{sc} is a scaling factor introduced to scale the mono-exponential PL decay to the scaling of $I_d(t)$ in Eq. S.14: $F_{sc} = I_d(t_0)$. For the modelling of the transfer efficiency, t_0 is set to 1×10^{-6} ns as zero cannot be used for practical calculation in the integral of Eq. S.14. Using a lower value for t_0 does not change the value of the efficiency in any case. The transfer efficiency takes values between 0 and 1. In terms of practical calculation, the integration is performed as a definitive integral using the python package scipy.integrate [29] and the boundaries were set to $t = 0.000\,001$–100 ns. Convergence of the integral was thoroughly tested with a wide range of input parameters covering the whole range of parameter values that were used for the figures.

Supplementary Note 2: Aggregate Formation in P3HT

In this note, we provide a more in-depth discussion of the investigation of chain conformation in thin films of the hole-transport material poly(3-hexylthiophene)(P3HT) [30, 31, 32]. Film morphology and aggregate formation within polymer films are commonly studied through analysis of absorption and PL spectra [33, 34, 35, 36, 37, 38].

The PL spectra of the P3HT films of different thicknesses on quartz and on $MAPbI_3$ are shown in Fig. 4 of the main text. The variation in thickness was achieved by changing spin speed in the fabrication routine from 2000 rpm to 6000 rpm in steps of 1000 rpm. The faster the spin speed, the thinner the polymer film was, which was confirmed with Dektak and absorption measurements (see Table S3). The films were excited at 460 nm with a fluence of 3–4 nJ cm^{-2}. A description of the emission spectra is given in the main text.

Normalized (at the absorption peak at 560 nm) and zoomed-in P3HT absorption components from P3HT:$MAPbI_3$:quartz samples are shown in Fig. 6. The absorption peak around 615 nm changes relative to the absorption peak at 560 nm for the films on $MAPbI_3$ substrates suggesting a change in aggregation with spin speed/film thickness for these films, which is however not the case for the P3HT films on quartz substrates. The change is least prominent for the thickest film spun at the lowest spin speed, which agrees with changes in the PL data, on which the discussion will be mainly focused. The absorption spectra presented here are obtained by subtracting the $MAPbI_3$ absorption spectrum and might therefore be more prone to error than the PL, which stems only from P3HT. Furthermore, a recent study highlighted that PL is more suited for the study of aggregation in P3HT thin films of different thickness than absorption [37].

P3HT is a widely researched polymer. Centered around a report by Brown et al. [39] on the relative weakness and temperature behaviour of the lowest energy feature in P3HT absorption spectra, the origin of the spectral features of P3HT have been subject of much debate and investigation [40, 41, 39, 42, 43, 44, 45, 46]. A widely used interpretation for the data is the model of weakly coupled H-aggregates, forming because of interchain interaction between the chromophores in thin films of P3HT, which was introduced and applied by

Spano and co-workers [44, 45]. For ideal H-aggregates, the transition to the ground state is dipole-forbidden and there is no 0-0 peak in the emission, but disorder can lead to an increase of relative intensity of the 0-0 peak to the 0-1 peak in emission and absorption [44, 34]. The model was extended to so called "HJ-aggregates" taking into account competition between interchain and intrachain interactions and therefore between the formation of H- and J-aggregates, respectively [46, 35]. Here, intrachain interaction characterizes electronic excitations that are delocalized along the polymer chains (through bond) whereas interchain interaction relates to the electronic excitations delocalized between chains (through space) [36]. Sample morphology is thereby assumed to play a decisive role and for thin films in which interchain coupling is dominant, the weakly coupled H-aggregate model offers a prevalent framework for data interpretation [46, 36].

Using the model of weakly interacting H-aggregates outlined above, we first compare the steady-state PL from P3HT films on a quartz substrate (dashed lines in Fig. 4 of the main text) with the PL from the films on $MAPbI_3$:quartz (solid lines in Fig. 4 of the main text). For the films on $MAPbI_3$:quartz we observe a blue-shift and an increase in 0-0 to 0-1 PL peak ratio corresponding to a weaker H-aggregate signature of suppressed 0-0 emission. We attribute the detected relative increase of the 0-0 peak of the PL from the films on $MAPbI_3$ to a decrease in interchain (through space) coupling, stemming from microstructural differences between those P3HT films [46, 35, 37]. A change in film morphology due to increased substrate surface roughness leading to less H-aggregate formation in P3HT films has been observed previously[47, 37]. Our observations agree more specifically with the work of Ehrenreich et al. [37] who compared PL lineshapes from a thin and a thick P3HT film on $MAPbI_3$ and attributed the difference to a P3HT film structure composed of an amorphous region with less interchain interaction formed at the interface to the rough metal halide $MAPbI_3$ and a more ordered region further away from this interface. A thin P3HT film is more dominated by PL from the amorphous region than a thick P3HT film. In agreement with this, we note that the PL features from our thickest (2000 rpm) P3HT film on $MAPbI_3$:quartz are most alike to the PL lineshape of the films on a smooth substrate while the thinner (3000 rpm to

6000 rpm) films show a more pronounced relative increase of the 0-0 PL peak and are more blue-shifted, which is a stronger signature of the amorphous phase at the $MAPbI_3$ interface.

So far, the focus was on the difference between P3HT films on a quartz substrate and on $MAPbI_3$:quartz. However, we observe additional differences in the PL spectra between films with varying thickness on a $MAPbI_3$:quartz substrate. Such a thickness dependence of the spectral lineshape of P3HT emission is observed only for P3HT films on the rough $MAPbI_3$:quartz and not for P3HT films on smooth quartz implying that further changes in aggregation are connected to the roughness of the $MAPbI_3$ surface.

One such difference in PL spectra is a decrease in relative 0-0 PL peak intensity with decreasing film thickness for all but the thickest P3HT:$MAPbI_3$:quartz film is. This is somewhat opposite to what would be expected from the explanation of film-thickness-dependent emission stemming either mainly from the amorphous layer close to $MAPbI_3$ (thinner films) or from a more ordered and H-aggregated layer on top (thicker films) and needs further discussion.

Before discussing this observation, we further analyse PL spectral changes of the P3HT:$MAPbI_3$:quartz films occurring with different film thickness: The relative intensity of the 0-0 peak decreases with decreasing film thickness and thus increasing spin speed. Within the framework of H-aggregates, this indicates that the interchain interaction is stronger in these thinner, faster spun-cast films [44, 34]. Additionally, we observe a change in relative intensity of the 0-2 to 0-1 PL peak in Fig. 4 of the main text with thickness and therefore with spin speed for the P3HT film on $MAPbI_3$: The 0-2 to 0-1 ratio is highest for the two thinnest films (5000 rpm and 6000 rpm), which also have a lower 0-0 to 0-1 peak ratio compared to the two intermediate films (3000 rpm and 4000 rpm).

For the discussion of these observations, we extended the framework to the HJ-aggregate model [46], which accounts for the interplay between inter- (through space) and intrachain (through the polymer bond) coupling. The strength of the two interactions can be assessed from the ratios of the 0-0 to 0-1 peak and of the 0-2 to 0-1 peak. It has been shown that

the 0-2 to 0-1 PL peak ratio is predominantly determined by intrachain (through bond) interactions and a decrease in relative peak intensity signifies stronger intrachain coupling [35]. The ratio of the 0-0 to 0-1 PL peak is an indication of the strength of interchain (through space) interaction as discussed above and still a main factor in the HJ-aggregate model [48]. In addition to that, when intrachain interaction is considered as well, the interplay between inter- and intrachain interaction influences the 0-0 to 0-1 peak ratio; stronger intrachain interaction leads to an increase in 0-0 to 0-1 peak ratio [35]. Applying this knowledge to our P3HT films on $MAPbI_3$, interchain coupling strengthens (0-0 to 0-1 PL ratio decreases) and intrachain coupling weakens (0-2 to 0-1 PL ratio increases) for the two thinnest films spun at highest spin speeds. At this point, it is however important to notice that the changes PL intensity ratios observed here are far weaker than the changes for example observed for P3HT nanofibers displaying mainly J-aggregate behaviour [49]. We therefore still consider our P3HT thin films spun-cast on $MAPbI_3$:quartz as predominantly exhibiting H-aggregation behaviour but in order to fully understand their PL spectral lineshape, intrachain interactions need to be taken into account through the HJ-aggregate model.

The impact of this discussion on film morphology and consequences for perovskite-based solar cells are illustrated in the main text.

References

[1] Ishida, H., Bünzli, J. C. & Beeby, A. Guidelines for measurement of luminescence spectra and quantum yields of inorganic and organometallic compounds in solution and solid state (IUPAC Technical Report) (2016).

[2] Trimpl, M. J. Charge-Carrier Recombination and Trapping Mechanisms in Metal Halide Perovskite Semiconductors. Master Thesis. Technische Universität München. (2019).

[3] NREL. Solar Spectra. https://www.nrel.gov/grid/solar-resource/spectra.html (accessed 2020-08-25).

[4] Tao, S. *et al.* Absolute energy level positions in tin- and lead-based halide perovskites. *Nat. Commun.* **10**, 1–10 (2019).

[5] Bolink, H. J., Coronado, E., Orozco, J. & Sessolo, M. Efficient Polymer Light-Emitting Diode Using Air-Stable Metal Oxides as Electrodes. *Adv. Mater.* **21**, 79–82 (2009).

[6] Bolink, H. J. *et al.* Inverted solution processable OLEDs using a metal oxide as an electron injection contact. *Adv. Funct. Mater.* **18**, 145–150 (2008).

[7] Sigma-Aldrich. https://www.sigmaaldrich.com/catalog/product/aldrich/445703 (accessed 2020-08-26).

[8] Saunders, B. R. & Turner, M. L. Nanoparticle-polymer photovoltaic cells (2008).

[9] Förster, T. Energiewanderung und Fluoreszenz. *Sci. Nat.* **6**, 166–175 (1946).

[10] Medintz, I. & Hildebrandt, N. (eds.) *FRET - Förster Resonance Energy Transfer* (Wiley-VCH Verlag GmbH & Co, Weinheim, 2014).

[11] Braslavsky, S. E. *et al.* Pitfalls and limitations in the practical use of Förster's theory of resonance energy transfer. *Photochem. Photobiol. Sci.* **7**, 1444–1448 (2008).

[12] Maksimov, M. & Rozman, I. On Energy Transfer in Solid Solutions. *Opt. Spectrosc. USSR* **12**, 337–338 (1961).

[13] Förster, T. Experimentelle und Theoretische Untersuchung des Zwischenmolekularen Übergangs von Elektroneneanregungsenergie. *Z. Naturforsch.* **4a**, 321–327 (1949).

[14] Förster, T. 10th Spiers Memorial Lecture. Transfer Mechanisms of Electronic Excitation. *Discuss. Faraday Soc.* **27**, 7–17 (1959).

[15] Hauser, M., Klein, U. K. A. & Gösele, U. Extension of Försters Theory of Long-Range Energy Transfer to Donor-Acceptor Pairs in Systems of Molecular Dimensions. *Z. Phys. Chem.* **101**, 255–266 (1976).

[16] Huber, D. L., Hamilton, D. S. & Barnett, B. Time-dependent effects in fluorescent line narrowing. *Phys. Rev. B* **16**, 4642–4650 (1977).

[17] Blumen, A. & Manz, J. On the concentration and time dependence of the energy transfer to randomly distributed acceptors. *J. Chem. Phys.* **71**, 4694–4702 (1979).

[18] Wolber, P. K. & Hudson, B. S. An analytic solution to the Förster energy transfer problem in two dimensions. *Biophys. J.* **28**, 197–210 (1979).

[19] Klafter, J. & Blumen, A. Fractal behavior in trapping and reaction. *J. Chem. Phys.* **80**, 875–877 (1984).

[20] Baumann, J. & Fayer, M. D. Excitation transfer in disordered two-dimensional and anisotropic three-dimensional systems: Effects of spatial geometry on time-resolved observables. *J. Chem. Phys.* **85**, 4087–4107 (1986).

[21] Farinha, J. P. & Martinho, J. M. Resonance Energy Transfer in Polymer Interfaces. In *4th Springer Series on Fluorescence Methods and Applications*, 215–256 (Springer Verlag Berlin Heidelberg, 2008).

[22] Farinha, J. P. & Martinho, J. M. Resonance Energy Transfer in Polymer Nanodomains. *J. Phys. Chem. C* **112**, 10591–10601 (2008).

[23] Yekta, A., Duhamel, J. & Winnik, M. A. Dipole-dipole electronic energy transfer. Fluorescence decay functions for arbitrary distributions of donors and acceptors: systems with planar geometry. *Chem. Phys. Lett.* **235**, 119–125 (1995).

[24] Valeur, B. & Berberan-Santos, M. N. *Molecular Fluorescence: Principles and Applications* (Wiley-VCH Verlag GmbH & Co, Weinheim, 2012).

[25] Campoy-Quiles, M., Alonso, M. I., Bradley, D. D. & Richter, L. J. Advanced Ellipsometric Characterization of Conjugated Polymer Films. *Adv. Funct. Mater.* **24**, 2116–2134 (2014).

[26] Tropf, L. *et al.* Influence of optical material properties on strong coupling in organic semiconductor based microcavities. *Appl. Phys. Lett.* **110**, 153302 (2017).

[27] Macdonald, E. K. & Shaver, M. P. Intrinsic high refractive index polymers. *Polym. Int.* **64**, 6–14 (2015).

[28] Ball, J. M. *et al.* Optical properties and limiting photocurrent of thin-film perovskite solar cells. *Energy Environ. Sci.* **8**, 602–609 (2015).

[29] Virtanen, P. *et al.* SciPy 1.0: Fundamental Algorithms for Scientific Computing in Python. *Nat. Methods* **17**, 261–272 (2020).

[30] Heo, J. H. *et al.* Efficient inorganic-organic hybrid heterojunction solar cells containing perovskite compound and polymeric hole conductors. *Nat. Photonics* **7**, 486–491 (2013).

[31] Zhou, P. *et al.* Efficient and stable mixed perovskite solar cells using P3HT as a hole transporting layer. *J. Mater. Chem. C* **6**, 5733–5737 (2018).

[32] Jung, E. H. *et al.* Efficient, stable and scalable perovskite solar cells using poly(3-hexylthiophene). *Nature* **567**, 511–515 (2019).

[33] Clark, J., Chang, J. F., Spano, F. C., Friend, R. H. & Silva, C. Determining exciton bandwidth and film microstructure in polythiophene films using linear absorption spectroscopy. *Appl. Phys. Lett.* **94**, 163306 (2009).

[34] Spano, F. C. The Spectral Signatures of Frenkel Polarons in H-and J-Aggregates. *Acc. Chem. Res.* **43**, 429–439 (2010).

[35] Paquin, F. *et al.* Two-dimensional spatial coherence of excitons in semicrystalline polymeric semiconductors: Effect of molecular weight. *Phys. Rev. B* **88**, 155202 (2013).

[36] Spano, F. C. & Silva, C. H- and J-Aggregate Behavior in Polymeric Semiconductors. *Annu. Rev. Phys. Chem.* **65**, 477–500 (2014).

[37] Ehrenreich, P. *et al.* H-aggregate analysis of P3HT thin films-Capability and limitation of photoluminescence and UV/Vis spectroscopy. *Sci. Rep.* **6**, 32434 (2016).

[38] Hildner, R., Köhler, A., Müller-Buschbaum, P., Panzer, F. & Thelakkat, M. π-Conjugated Donor Polymers: Structure Formation and Morphology in Solution, Bulk and Photovoltaic Blends. *Adv. Energy Mater.* **7**, 1700314 (2017).

[39] Brown, P. J. *et al.* Effect of interchain interactions on the absorption and emission of poly(3-hexylthiophene). *Phys. Rev. B* **67**, 064203 (2003).

[40] Sakurai, K. *et al.* Experimental determination of excitonic structure in polythiophene. *Phys. Rev. B* **56**, 9552–9556 (1997).

[41] Jiang, X. M. *et al.* Spectroscopic Studies of Photoexcitations in Regioregular and Regiorandom Polythiophene Films. *Adv. Funct. Mater.* **12**, 587–597 (2002).

[42] Koren, A. B., Curtis, M. D., Francis, A. H. & Kampf, J. W. Intermolecular Interactions in π-Stacked Conjugated Molecules. Synthesis, Structure, and Spectral Characterization of Alkyl Bithiazole Oligomers. *J. Am. Chem. Soc.* **125**, 5040–5050 (2003).

[43] Kobayashi, T. *et al.* Coexistence of photoluminescence from two intrachain states in polythiophene films. *Phys. Rev. B* **67**, 205214 (2003).

[44] Spano, F. C. Modeling disorder in polymer aggregates: The optical spectroscopy of regioregular poly(3-hexylthiophene) thin films. *J. Chem. Phys.* **122**, 234701 (2005).

[45] Clark, J., Silva, C., Friend, R. H. & Spano, F. C. Role of Intermolecular Coupling in the Photophysics of Disordered Organic Semiconductors: Aggregate Emission in Regioregular Polythiophene. *Phys. Rev. Lett.* **98**, 206406 (2007).

[46] Yamagata, H. & Spano, F. C. Interplay between intrachain and interchain interactions in semiconducting polymer assemblies: The HJ-aggregate model. *J. Chem. Phys.* **136**, 184901 (2012).

[47] Halászová, S., Váry, T., Nádašdy, V., Chlpík, J. & Cirák, J. Effect of Substrate Roughness on Photoluminescence of Poly(3-Hexylthiophene). In *AIP Conference Proceedings*, vol. 1996, 20051 (2018).

[48] Pereira, V. S., Leal, L. A., Ribeiro Junior, L. A. & Blawid, S. Inferring changes in π-stack mobility induced by aging from vibronic transitions in poly(3-hexylthiophene-2,5-diyl) films. *Synth. Met.* **247**, 276–284 (2019).

[49] Niles, E. T. *et al.* J-Aggregate Behavior in Poly-3-hexylthiophene Nanofibera. *J. Phys. Chem. Lett.* **3**, 259–263 (2012).
